# Discerning mechanistically rewired biological pathways by cumulative interaction heterogeneity statistics

**DOI:** 10.1038/srep09634

**Published:** 2015-04-28

**Authors:** Travis B. Cotton, Hien H. Nguyen, Joseph I. Said, Zhengyu Ouyang, Jinfa Zhang, Mingzhou Song

**Affiliations:** 1 Department of Computer Science, New Mexico State University, NM 88003, Las Cruces, USA; 2 Department of Plant and Environmental Sciences, New Mexico State University, NM 88003, Las Cruces, USA

## Abstract

Changes in response of a biological pathway could be a consequence of either pathway rewiring, changed input, or a combination of both. Most pathway analysis methods are not designed for mechanistic rewiring such as regulatory element variations. This limits our understanding of biological pathway evolution. Here we present a *Q*-method to discern whether changed pathway response is caused by mechanistic rewiring of pathways due to evolution. The main innovation is a cumulative pathway interaction heterogeneity statistic accounting for rewiring-specific effects on the rate of change of each molecular variable across conditions. The *Q*-method remarkably outperformed differential-correlation based approaches on data from diverse biological processes. Strikingly, it also worked well in differentiating rewired chaotic systems, whose dynamics are notoriously difficult to predict. Applying the *Q*-method on transcriptome data of four yeasts, we show that pathway interaction heterogeneity for known metabolic and signaling pathways is indeed a predictor of interspecies genetic rewiring due to unbalanced TATA box-containing genes among the yeasts. The demonstrated effectiveness of the *Q*-method paves the way to understanding network evolution at the resolution of functional biological pathways.

Modular and hierarchical orderings of molecules in a cell give rise to the biochemical mechanisms underlying functionally distinct biological pathways. Chemical reactions and physical interactions among these molecules govern observed pathway response. We call genetic or epigenetic modifications to the connections among molecules mechanistic rewiring. Both mechanistic rewiring and changed upstream input, though two fundamentally different causes, can elicit divergent pathway responses. Under the overarching goal to understand how evolution may have rewired a biological pathway, we aimed at discerning whether a changed pathway response is likely a result of mechanistic modifications within the pathway or changed upstream input to the pathway. Although other studies linked rewired gene interactions, e.g., TATA box variation, to gene expression variability among related species^1^, methods for the rewiring analysis of functional pathways are lacking.

Due to its cumulative nature, pathway analysis is more effective for interpretation of biological functions than single-gene approaches at modest effect and sample sizes. Most pathway analysis methods, e.g., GSEA^2^, accrue expression change of genes in a pathway. Some generalized statistical tests on collective ranks of genotype-phenotype associations of all pathway genes^3^. They are, however, insensitive to pathway topology.

Available pathway topology databases^4^,^5^ prompted methods to exploit such information. NetGSA^6^ used a latent variable model, estimated by a mixed linear model, to incorporate gene interactions in a pathway. SPIA^7^ also used a static linear pathway impact model^8^ based on gene interactions. A recent review^9^ listed 22 topology-based pathway analysis methods which were not designed to differentiate whether changed dynamics are a consequence of pathway rewiring or changed pathway input. Their resulting top-ranked pathways are thus not necessarily most mechanistically rewired.

ScorePAGE^10^ and Gene Set Co-expression Analysis (GSCA)^11^ adopted cumulative pairwise correlation scores to evaluate gene interactions in a pathway, though GSCA also includes those not in the pathway topology. ScorePAGE was not specifically designed for comparative pathway analysis across multiple experiments. GSCA evaluates a divergence index calculated by the summation of absolute differential correlation and is highly relevant to pathway rewiring. These methods are however not equipped for nonlinear or dynamical pathway representations.

After reviewing 33 pathway analysis methods, Khatri et al^12^ posed six challenges to this research area. We present the *Q*-method to meet three of them. Specifically, we address the third challenge, the annotation challenge: “missing condition- and cell-specific information” by adapting an annotated pathway topology to context-specific data, instead of using the topology exactly, to allow pathway rewiring. Our main design consideration overcomes methodological challenge 2 “inability to model and analyze dynamic response,” by adopting nonlinear dynamical system models (DSMs). Responding to methodological challenge 3 “inability to model effects of an external stimuli,” we distinguish whether observed dynamic changes are caused by external stimuli or intrinsic pathway rewiring.

The *Q*-method discerns mechanistically rewired pathways by comparative dynamical system modeling. We consider a DSM to be *conserved* if it has the same parameters or coefficients with the original model but the variables in the DSM are not required to have the same initial values. On the other hand, a *rewired* or *differential* DSM must have different parameters from the original model, regardless of the initial values for model variables. Given a super-pathway topology, we partially reconstruct the active interactions in this pathway. All active interactions constitute one homogeneous and two heterogeneous DSMs for a pathway under two conditions. If the heterogeneous models are significantly supported by observed data, the pathway is declared to undergo a mechanistic rewiring, or differential; otherwise, it is conserved. In this paper, we reserve the word *differential* for mechanistic differences and use the word *divergent* for dynamical response differences. Using 12 modes from the BioModels database^13^, the *Q*-method detected pathway rewiring more effectively than GSCA in most cases. Chaotic dynamical systems are increasingly recognized in molecular biology^14^,^15^, and the *Q*-method successfully differentiated rewired Lotka-Volterra predator-prey models. Using interspecies yeast transcriptomes, the *Q*-method predicted rewired pathways consistent with TATA box disparity, painting rewired mechanisms among four yeast species. When the rate of change for biological quantities can be reliably estimated, our results suggest broad applicability of the *Q*-method for detecting pathway rewiring in dynamic biological networks at the mechanistic level.

## Methods

### Overview of the *Q*-method

**Figure 1:**
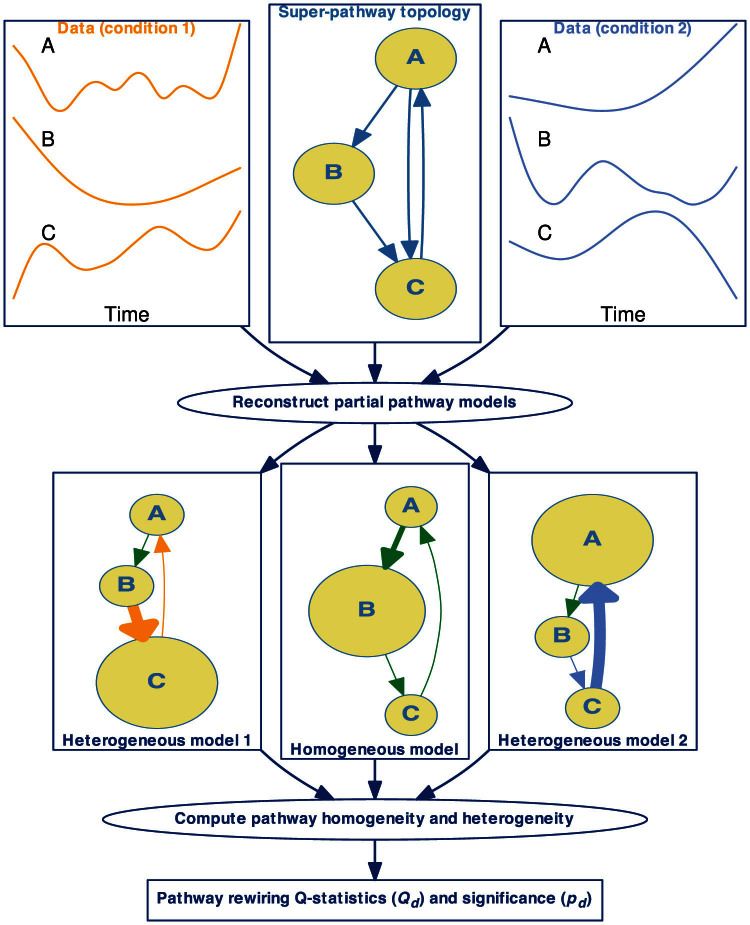
Overview of the *Q*-method for detecting pathway rewiring. The input requires observed time course data from a pathway under multiple conditions. The input also includes a super-pathway topology, which can be either retrieved from a pathway topology database or causally inferred from the observed data. The first main step is to reconstruct partial pathway models – heterogeneous differential equation models specific to each condition. The strength of interaction heterogeneity is calculated for each node. Also a homogenous differential equation model is created from data pooled from all conditions. The second main step is to compute pathway interaction heterogeneity and homogeneity statistics and their statistical significance. The final output is thus a set of statistics, including *Q_d_* and *p_d_* measuring the strength and statistical significance of mechanistic pathway rewiring. The thickness of edges and the size of nodes indicate the strength of rewiring in the heterogeneous models; they indicate conservation in the homogeneous model.

The *Q*-method tests whether a pathway is mechanistically rewired across two conditions or species, as summarized in Fig. 1. It also infers causal DSMs that best explain the observed time course data for each condition. The input includes a given super-pathway topology and observed time course data *D*_1_ and *D*_2_ under each of two conditions. Partial pathway reconstruction is first performed to infer active interactions in the super-pathway topology. This partial reconstruction step can be done using many network inference methods and here we use dynamical system modeling to capture rate of change information. It is *partial* because only those inferred *active* interactions constitute one homogeneous and two heterogeneous DSMs for a pathway under two conditions. The two heterogeneous DSMs can be used to determine rewired interaction strength or topology across conditions by statistically meaningful differences in model parameters. Three *Q*-statistics and their statistical significance are calculated to characterize pathway interaction homogeneity (*Q_c_*, *p_c_*), heterogeneity (*Q_d_*, *p_d_*), and total interaction strength (*Q_t_*, *p_t_*). By pathway interaction heterogeneity/homogeneity, we measure the cumulative difference/similarity in the manner that pathway elements interact across conditions or species. By total interaction strength, we measure the overall dynamical activity of a pathway. For example, a pathway already reaching a steady state in all experimental conditions will have a low total interaction strength. The overall null hypothesis is that the pathway is not rewired across conditions. Statistical significance of the *Q*-statistics determines, as the output, whether changed pathway response can be explained by mechanistically rewired DSMs under each condition. The *Q*-method software is freely available to non-commercial organizations at http://www.cs.nmsu.edu/~joemsong/software/Q-method.

### Representing pathways by dynamical system models

We represent pathway mechanisms by DSMs. The key advantage of DSM is its ability to track rate of change in a dynamical system. It is widely used in mathematical biology but disproportionally under-explored for pathway analysis. With rate-of-change information, we are theoretically equipped to distinguish the causes for observed differences in dynamics between biological systems. Each node on a pathway is mapped to a variable in a DSM. Via a system of additive nonlinear ordinary differential equations (ODEs), a DSM quantifies the rate of change for each child variable as a function of its parent variables. Each ODE uses a linear combination of linear, quadratic, and sigmoidal terms as functions of parent variables. Linear terms delineate the simplest possible interactions; quadratic terms can capture combinatorial effects; and sigmoidal terms approximate transcription activation kinetics^16^ and capture basal activation, switch-like or partial linear response, and saturation. Let a DSM contain *p* variables, each representing the abundance of a molecule. Let *x_i_*(*t*) be the value of variable *i* at time *t*. Let *y_i_*(*t*) be its rate of change. Let 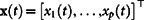 be a vector representing the state of the system at time *t*. Let 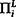, 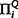, and 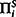 be the set of parent variable indices giving rise to linear, quadratic, and sigmoidal terms for variable *i*. 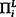 and 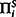 are integer sets and 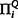 is a set of integer pairs. Now we can write the ODE for child variable *i* as
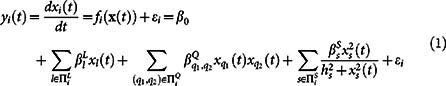
where *β*_0_ is a constant, 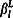 is the coefficient of the linear term involving variable *l*, 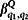 coefficient of quadratic term involving variables *q*_1_ and *q*_2_, and 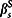 coefficient of the sigmoidal term involving variable *s*, and 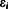 is a noise term. *h_s_* is the value of variable *s* producing half target occupation, i.e. when *x_s_*(*t*) = *h_s_*, the term 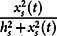 is equal to 0.5. *f_i_* is the functional part of the model. For clarity purpose, we omitted the subscript *i* for all coefficients, or parameters, though they are indeed child specific.

### Partial pathway reconstruction

We partially reconstruct four pathway DSMs and compute residuals using the given super-pathway topology as a superset of all possible interactions. Partial refers to the fact that a reconstructed DSM contains only a subset of active interactions from the super-pathway topology. Directed-graph representations of pathways can be extracted from Kyoto Encyclopedia of Genes and Genomes (KEGG)^17^, BioModels, or other resources. Specifically, the first DSM uses data under condition 1, the second with data under condition 2, the third with data pooled from both conditions, and the fourth is a null model with only the constant term *β*_0_ from the pooled data. Pooled data are obtained by combining the data sets from all conditions.

We apply an *F*-test method to reconstruct the first three pathway DSMs by estimating the parameters in the DSMs (see Supplementary Text 3.1 for full detail of model estimation). The choice of parents in 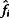, a best estimate of *f_i_*, is constrained to be a subset of all possible parents of node *i* as specified in the super-pathway topology. We remark here that this first partial reconstruction step can be accomplished using alternative causal network inference methods. The super-pathway topology is relevant only if it indeed covers all possible interactions expected in a network. Otherwise, a complete data-driven approach is more appropriate when sufficient experimental data are available.

We use the term *D*_1_ to refer to all information under condition 1, including state vector **x**^(1)^(*t*), derivative 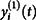 of variable *i*, the number of time points sampled *n*_1_ occurring non-uniformly over time at *T*^(1)^ = {*t_k_*|*k* = 1, ¼, *n*_1_}, observed data **x**^(1)^(*t_k_*) at *t_k_*, and estimated derivative 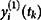 using penalized smoothing splines implemented in the R package pspline. Symmetrically, we define *D*_2_ for condition 2, including **x**^(2)^(*t*), 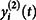, *n*_2_, *T*^(2)^, **x**^(2)^(*t_k_*), and 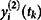.

We first obtain two heterogeneous pathway models for each condition. By comparative dynamical system modeling^18^, we partially reconstruct the best models simultaneously for both conditions sharing a subset of interactions from the given super-pathway topology. Interactions in the subset are active under at least one condition. For condition 1, the estimated pathway DSM *P*^(1)^ is
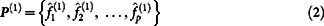
where 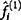 is the estimated model with *m*^(1)^(*i*) parameters for variable *i*. The model complexity, or number of parameters, of *P*^(1)^ is 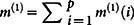. Thus, the residual sum of squares (*RSS*) for variable *i* is
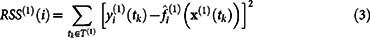
For condition 2, the DSM model is
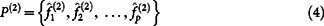
with model complexity 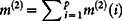 and
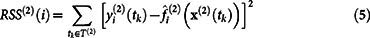
We call *P*^(1)^ and *P*^(2)^ heterogeneous models because their parameters are optimized for each condition.

Novelty of our pathway analysis builds on two additional models, a homogeneous model and a pooled null model, to be used to examine pathway homogeneity and also as a reference for pathway heterogeneity. With pooled data 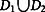 and the sub-graph obtained when determining *P*^(1)^ and *P*^(2)^, we get the pooled pathway DSM:
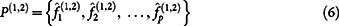
where 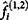 for variable *i*, estimated using the pooled data, has *m*^(1,2)^(*i*) parameters. We call *P*^(1,2)^ the homogeneous model as it fits to data under both conditions. The model has complexity 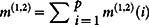 and *RSS* for variable *i*
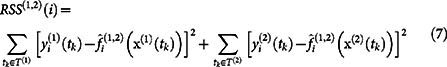


We write the pooled null model of the pathway by
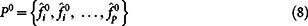
where 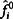 is a constant function whose value is the time average of observed data *y_i_*(*t_k_*) for variable *i* over pooled sample in 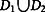. The model complexity of 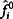 is *m*^0^(*i*) = 1. The model complexity of *P*^0^ is thus 
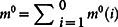
 with *RSS* for variable *i*
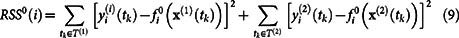


With all *RSS*'s defined, we backtrack to give the criterion used to reconstruct the pathway models. To capture total interaction strength for both conditions for variable *i*, we use the *F*-statistic
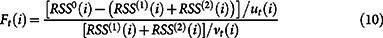
where *u_t_*(*i*) = *m*^(1)^(*i*) + *m*^(2)^(*i*) − *m*^0^(*i*) and *v_t_*(*i*) = *n*_1_ + *n*_2_ − (*m*^(1)^(*i*) + m^(2)^(*i*)) are the numerator and denominator degrees of freedom. The parent variables involved in 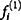 and 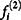 were selected to minimize the *p*-value of *F_t_*(*i*).

In summary, partial pathway reconstruction produces four DSM models with *RSS*'s for comparative pathway analysis. In contrast to full network reconstruction without pathway topology, partial pathway reconstruction is efficient with a greatly reduced search space of interactions spanned only by sub-graphs of the given super-pathway topology. As pathway conditions and cellular contexts are often not encoded in a database, partial reconstruction tailors a pathway to observed data. This strategy is effective when a pathway topology is inclusive of interactions known to occur in some molecular contexts but not necessarily all active in the current experiment. Although the pathway DSMs share a subgraph topology, an interaction or edge in the subgraph can be considered deleted in a condition if the DSM parameter corresponding to that interaction approaches zero under the condition.

### *Q*-statistics for pathway homogeneity and heterogeneity

Changed pathway response can be caused by various modifications to biological mechanisms. Mutations in promoter sequences, the presence or absence of enhancer or silencer regions, mutations in general transcription factors, chromatin remodeling, or changes in upstream promoter regions of genes may all alter dynamics of regulatory pathways. Parameters in heterogeneous pathway models *P*^(1)^ and *P*^(2)^ may differ numerically due to noisy observations even when the underlying systems are identical. Direct numerical comparison of the two models would lead to high false-positive rates for differential pathways. Instead, using the four pathway models and their residuals, we develop a statistically reliable framework to evaluate pathway homogeneity and heterogeneity across conditions.

Homogeneity is the improvement of homogeneous model *P*^(1,2)^ over the pooled null model *P*^0^. We compute the overall homogeneous *RSS_hom_* of *P*^(1,2)^, indicating its absolute goodness-of-fit to the data, by adding the individual *RSS*'s for each variable
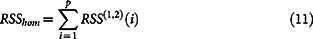
Heterogeneity is the improvement using two models *P*^(1)^ and *P*^(2)^ over a single pooled model *P*^(1,2)^. We measure the overall heterogeneous *RSS_het_* of *P*^(1)^ and *P*^(2)^, also indicating their absolute goodness-of-fit to the data, by
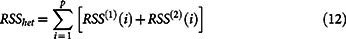
To account for a pathway being active in neither condition, we also consider *RSS_null_* of the pooled null model *P*^0^
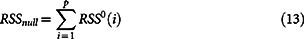


Although an *RSS* indicates model goodness-of-fit, it is unfair to compare them directly because of unequal model complexity. Instead, we compute their *RSS* differences normalized by degrees of freedom as in an *F*-test. To capture pathway interaction heterogeneity across conditions, we measure the normalized difference between *RSS_hom_* and *RSS_het_* by
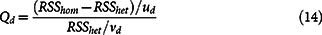
where *u_d_* = *m*^(1)^ + *m*^(2)^ − *m*^(1,2)^ and *v_d_* = *n*_1_ + *n*_2_ − (*m*^(1)^ + *m*^(2)^) are the numerator and denominator de grees of freedom. A high *Q_d_* value for pathway interaction heterogeneity provides strong evidence for a pathway to have been rewired across experimental conditions.

To capture pathway interaction homogeneity across conditions, we measure the normalized difference between *RSS_null_* and *RSS_hom_* by
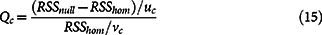
where *u_c_* = *m*^(1,2)^ − *m*^0^ and *v_c_* = *n*_1_ + *n*_2_ − *m*^(1,2)^ are the numerator and denominator degrees of freedom. A high *Q_c_* value for pathway interaction homogeneity suggests that a pathway has a consistent wiring behavior across conditions.

To capture total pathway strength for both conditions, we measure the normalized difference between *RSS_null_* and *RSS_het_* by
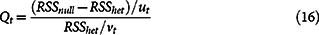
where *u_t_* = *m*^(1)^ + *m*^(2)^ − *m*^0^ and *v_t_* = *n*_1_ + *n*_2_ − (*m*^(1)^ + *m*^(2)^) are the numerator and denominator degrees of freedom. A high *Q_t_* value for overall pathway involvement suggests strong interaction patterns are observed for the pathway regardless of conserved or differential.

### The pathway decomposition rule

Pathway homogeneity *Q_c_*, heterogeneity *Q_d_*, and total strength *Q_t_* mathematically constrain each other. From their definitions in Eqs (14), (15), and (16), we obtain
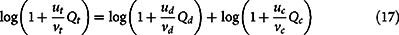
We call this relationship the *pathway interaction decomposition rule*, extending the decomposition rule for a single interaction across conditions^18^. In this rule, the total pathway interaction strength is decomposed to pathway homogeneity and heterogeneity. Therefore, knowing two of the three statistics entails the third. Given the total interaction strength, homogeneity and heterogeneity counterbalance each other. Less obviously, two heterogeneous systems can still have none-zero homogeneity, which allows differing systems to share certain commonality. For example, a transcription factor can regulate a target gene differentially due to genome evolution between two species. The binding affinity in one species can be higher than the other species, though resulting in positive regulation in both cases. A pathway containing such transcription factors can thus exhibit high heterogeneity due to the changed binding affinity and also certain non-zero homogeneity due to the consistent positive regulatory effect.

### Deciding pathway rewiring by statistical significance

Based on statistical significance of observed pathway homogeneity and heterogeneity, we decide whether a pathway is conserved or differential. Under standard normality assumptions for the *F*-test as in regression analysis, *Q_c_*, *Q_d_*, and *Q_t_* follow asymptotic *F*-distributions with their respective numerator and denominator degrees of freedom under the null hypotheses^19^. The resulting *p*-values *p_c_*, *p_d_*, and *p_t_* are statistical significance of pathway homogeneity, heterogeneity, and total interaction strength.

To address the potential violation of the normality assumption for the residuals, dependent nodes in a pathway, or evaluation of multiple pathways, we use a permutation test to adjust statistical significance of *Q_d_* and *Q_c_*. We randomly sample nodes in the entire system to form new pathways and permute their conditions. The permuted 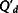 and 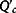 values give empirical null distributions, and statistical significance *p_d_* and *p_c_* of the originally observed *Q_d_* and *Q_c_* are determined by the fraction of permutations with 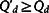 and 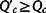.

Finally, if pathway heterogeneity is significant at a given type I error *α*-level, i.e., *p_d_* ≤ *α*, the pathway is differential regardless of homogeneity; the pathway is conserved if homogeneity is significant at *p_c_* ≤ *α* and the heterogeneity is insignificant (*p_d_* > *α*); otherwise, there is insufficient evidence to declare a differential or conserved pathway across conditions.

## Results

### Advantage of *Q*-method over its alternatives

To evaluate the performance of *Q*-method, we simulated data from 12 curated dynamic models (Table 1) in the BioModels database^13^ and compared it with the GSCA method. These models are some of the best representations of classical biological processes with complex dynamics supported by a large amount of experimental data. They are composed of nonlinear ODEs in different forms from our additive nonlinear model in Eq. (1). As it is rarely practical to know the correct mathematical forms for a real biological system, we test if our method, by approximating the ODEs, answers the differential/conserved question sufficiently well. Each model is treated as a pathway. We simulated biological variations by changed coefficients in the differential equation models. For example, protein-binding affinity could be changed because of mutations in the protein amino acid sequence and this leads to a different affinity constant in a rewired model. Another example is that changed protein-DNA binding affinity due to sequence variation in the binding motif can also be reflected as a changed coefficient in the transcription regulation kinetics.

We generated ground truth of conserved and differential models from original models in BioModels database^13^. A conserved model is the same with an original model but with a changed initial state, which can have a dramatic effect on the system response. The initial value of a variable was changed by adding a random number uniformly distributed in [*L*, *U*] to the original initial state. *L* and *U* are determined by model sensitivity to the initial state. Too much change can break a relationship between variables due to numerical instability. To create a differential model, parameter values in the original model are multiplied by a random number normally distributed with mean *μ* and standard deviation *σ*. To avoid numerical instability, *μ* and *σ* vary depending on model sensitivity to parameters. To cover diverse initial states and parameters, we generated 100 conserved and 100 differential models for each original model. Given a model and its initial state, we simulated time courses and added noises at signal-to-noise ratios (SNRs) of 100, 20, 10, or 0 dB. The time courses were generated by solving ODEs in each model using R package deSolve^20^, and sampled at 100 time points, uniformly spaced with a model-dependent time interval. The perturbation values for each model are described in Table 1, and the runtime of the simulation study on each model is given in Table 2.

Given a pair of time courses and model (pathway) topology, the *Q*-method decides if they are from a pair of conserved (negative) or differential (positive) models based on *p_d_* and *α*. The decision is compared with the ground truth to tabulate the numbers of false positives and true positives at different *α*-levels to produce a receiver operating characteristic (ROC) curve.

**Figure 2:**
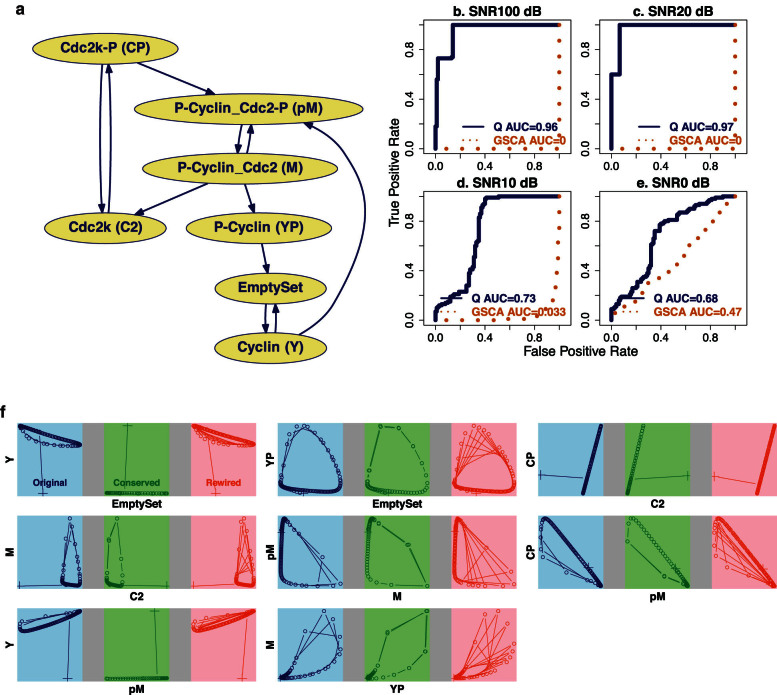
*Q*-method remarkably outperformed differential correlation in detecting rewired cell division cycle models. Fundamental limitations are inherent with differential correlation based methods on tracking trajectory shape but not rate of change. Nonlinear dynamic behavior in rewired cell division cycle model is captured by the *Q*-method but not GSCA. (a) The topology of the original cell division cycle model (BioModels ID BIOM0000000005) used in the pathway analysis. (b–e) The four plots show the ROC curves and area under the curves (AUCs) of the *Q*-method (blue solid lines) and GSCA (orange dots) detecting rewiring in 100 conserved and 100 differential/rewired models based on the original cell division cycle model at SNRs of (b) 100, (c) 20, (d) 10, and (e) 0 dB. The true positive rate is the ratio of correctly detected rewired models among all truly rewired models. The false positive rate is the ratio of incorrectly detected rewired models among all truly conserved models. Although the performance of Q-method deteriorates as noise increases, the increasing trend of GSCA's AUCs suggests that it is inadequate to keep track of mechanistic differences in this dynamical system. (f) Phase planes of the original (blue), conserved (green), and rewired (red) models are shown for each pair of directly interacting molecules. “+” marks the initial state. Phase planes depict interactions among Cdc2, Cyclin, and their complexes. Between the original and the conserved models, many interactions such as CP → C2 show a substantial change in the shape of the trajectory, but less from the original to rewired model. Differential correlation relies on superficial difference/similarity of trajectory footprint, which is neither sufficient or necessary for differential/conserved models. *Q*-method tracks the rate of change to resolve this issue.

We first demonstrate the performance of the *Q*-method on differentiating a rewired cell division cycle model^21^. This model describes dynamical interactions of cdc2 and cyclin in cell devision cycle with cubic terms not covered by our ODEs. The topology of the model is shown in Fig. 2a and the rewiring parameters of this model are given in Table 1. Here an edge incident upon the EmptySet represents degradation of a molecule and edge from the EmptySet represents synthesis of a molecule. Depending on activity of the protein complex maturation promoting factor (MPF, or Cyclin_Cdc2-P (M) in Fig. 2a), this pathway operates between an oscillation and an excitable domain. Full detail of chemical reactions in this model can be retrieved as BIOM0000000005 from the BioModels database^13^. After applying the *Q*-method on time course data simulated from 100 conserved and 100 rewired versions of this model, we plot the ROC curves (Fig. 2b–e) at four SNRs and show that the *Q*-method is effective even at a high level of noise when SNR is 0 dB. Supplementary Figures S1 to S4 further show ROC curve performance of our method at four SNRs for all 12 BioModels, which cover cell division cycle, circadian oscillations, intracellular calcium oscillations, glycolysis, synthetic gene-metabolic oscillator, and mitosis.

To demonstrate the advantage of *Q*-method, we also applied the original code of GSCA to exactly the same data in this simulation study. By summing up differential correlation between all time-course pairs of elements on a pathway across two conditions, GSCA calculates a dispersion index to determine differential pathways. Supplementary Figures S1 to S4 overlay the resulting ROC curves with those of the *Q*-method at all four SNRs – the ROC curves of GSCA are qualitatively less satisfactory in most of the models at most noise levels than the *Q*-method.

Table 3 summarizes average areas under the curve (AUCs) at different noise levels, demonstrating overwhelming strength of the *Q*-method over GSCA. The *Q*-method outperformed GSCA in AUC in 48 ROC curves and it performed slightly worse in only 7. The two methods are comparable in several models as illustrated in Supplementary Figures S5 and S6. Nevertheless, pair-wise differential correlation, used by GSCA, can be ineffective in approximating change in linear^18^ or nonlinear interactions.

The phase planes for the cell division cycle model in Fig. 2f illustrate the problems with differential-correlation-based approaches leading to underperformance as exhibited in Fig. 2b–e. With different initial states applied to the original (blue) and the conserved (green) models, the systems show divergent responses in all three variables. For example, the phase plane between Y and pM would apparently confuse a differential-correlation-based approach to determine strongly changed correlation coefficients between the original and the conserved models, despite the same differential equations were used to generate the trajectories. Additionally, the original (blue) and the rewired (red) models almost always show similar responses in this simulation, e.g., in the phase plane between M and C2. This would lead to small differential correlation and miss contribution to rewiring from this pair of molecules. In fact, none of the 8 phase planes demonstrates a strong difference in correlation coefficients between the rewired and the original models. This explains the far worse performance of GSCA for this model shown in Fig. 2b–e. In contrast, the *Q*-method tracks deeper into the rate of change and is thus more resistant to superficial differences or similarities in trajectory shape.

**Figure 3:**
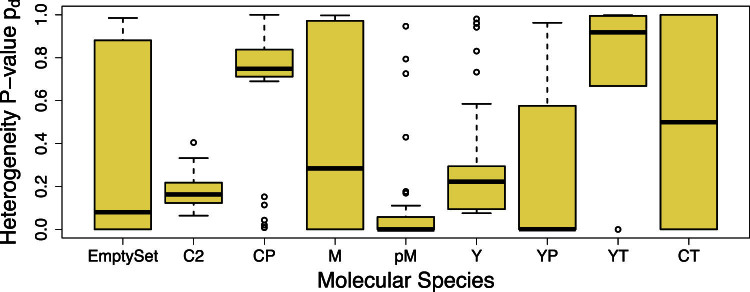
Individual molecular species respond to pathway rewiring differently in conserved and rewired cell cycle division models. The box plot shows the detected range of interaction heterogeneity *p*-value *p_d_* of individual molecular species in 15 conserved and 15 rewired models at an SNR of 100. A smaller *p*-value is associated with stronger interaction heterogeneity. In addition to the seven species defined in Fig. 2a, two implicit mathematical molecular species are included in the model: YT and CT representing total cyclin and total cdc2, respectively. The molecular species (M, CT) with full range in [0,1] of *p*-values are the most sensitive to rewiring; those (CP, YT) with *p*-values towards 1 are not responsive to the rewiring; and those (EmptySet, C2, pM, Y, YP) with *p*-values towards 0 are overly sensitive to rewiring and are thus subject to noise influence.

A rewired pathway does not require all molecular species in the system to undergo differential interactions. The *Q*-method thus also reports the interaction heterogeneity *p*-value *p_d_* for each individual molecular species. This *p_d_* value of a molecule is the statistical significance of *Q_d_* computed when the pathway includes only this molecule and its immediate parents. The box plots of the interaction heterogeneity *p*-values in the cell division cycle model under the SNR of 100 are shown in Fig. 3. This will allow one to select those molecules most responsive to pathway rewiring. The MPF, the protein complex M formed by Cyclin and Cdc2-P, shows a full heterogeneity range on data from both conserved and rewired models. This indicates that it is a very important molecular species responding robustly to rewiring. In fact, one of the most important kinetic rate constant *k*_4_ determines how fast the M complex is formed by dephosphorylation of Cdc2 in the pM complex. The value of *k*_4_ directly controls the state of cell cycle division: the system moves from the excitable domain to the oscillatory domain as *k*_4_ increases. In this manner, one can infer potential genotype modifications, e.g., *k*_4_, through the most responsive phenotypes, e.g., the abundance of the M complex.

### Differentiating chaotic dynamical systems

Chaotic gene expression oscillation has been observed in pluripotent cells, though lost in differentiated cells^15^. Chaos is also believed to maintain stable gene expression patterns in robust mutants^14^. A tiny change to initial conditions of a chaotic system can have a dramatic effect on its dynamics. This makes predicting their behavior challenging. Chaos is, however, deterministic. For this reason, we contemplate that for *given observed* chaotic dynamic behavior, distinguishing conserved or differential underlying chaotic systems is possible.

**Figure 4:**
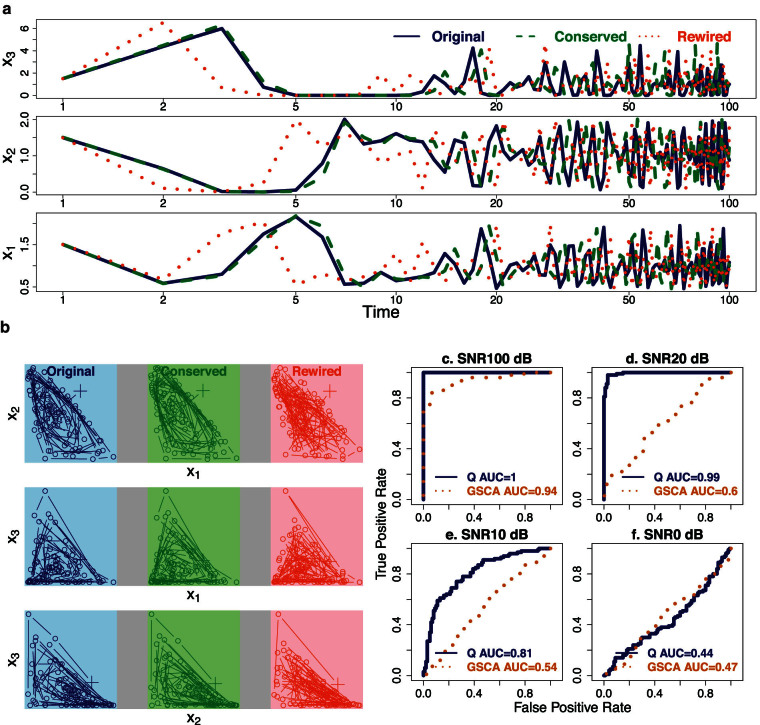
Advantage of the *Q*-method on differentiating chaotic dynamical systems. Responses can be divergent with both conserved and rewired versions of a 3-variable chaotic Lotka-Volterra model. (a) The time courses are almost noise-free at an SNR of 100 dB. Reponses of the rewired model (orange dots) diverged from the original (blue solid lines) at the time of about 3; the time course of the conserved model (green dashes) is similar to the original at early time points but also diverged around time 15. The original and the rewired LV models start at the initial state of (1.500, 1.500, 1.500). The rewired model has changed parameters from the original model. The conserved model starts at (1.504, 1.510, 1.509). (b) Phase planes demonstrate chaotic dynamic behavior of the 3-variable Lotka-Volterra model. Each group of three phase planes in one grey box is for one pair of variables in the rewired (red), conserved (green), and original (blue) models. “+” marks the initial state. Most trajectories appear to differ substantially. This presents a major challenge to most pathway analysis methods not based on rate of change. (c–f) The four plots show ROC curves and their AUCs of the *Q*-method and GSCA on comparing the 100 rewired and 100 conserved Lotka-Volterra models at SNRs of (c) 100, (d) 20, (e) 10, and (f) 0 dB. The true positive rate is the ratio of correctly detected rewired models over all truly rewired models. The false positive rate is the ratio of incorrectly detected rewired models over all truly conserved models. Although the performance of both methods deteriorate as noise increases, differences in the AUCs again suggest a remarkable advantage of *Q*-method over GSCA.

To understand the *Q*-method performance on chaotic systems, we used a 3-variable Lotka-Volterra (LV) model of predator-prey systems that can exhibit chaotic behavior with certain parameters. The LV model is defined by
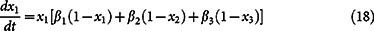

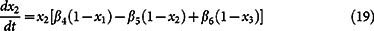

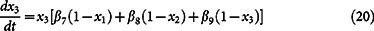
where *x*_1_, *x*_2_, and *x*_3_ are variables and *β*_1_ to *β*_9_ are model parameters. The LV model, originated from modeling biochemical reactions, has been widely used to model competitive relationships in ecology and also economics. Its dynamics can exhibit point attractors, limited cycles, and chaos, depending on the parameter choices. From the original model, we also created conserved and differential models as ground truth and then simulated data from all models with noise added. Figure 4a shows dynamics of the original LV model in its chaotic mode; a conserved LV model with a changed initial state; and a rewired LV model with changed parameters but the same initial state with the original model. The time courses suggest that the rewired model diverged before the 3rd time point and the conserved model also diverged but at around time point 15. The phase planes of the LV model in Fig. 4b suggest that the differential correlation measure will not be much discriminative between the rewired and conserved models, because the similar footprints of the trajectories indicate the correlation coefficients among the three models are numerically close.

Applying both the *Q*-method and GSCA on the simulated data, we obtain ROC curves at four noise levels in Fig. 4c–f. At each noise level for each model, we generated 100 noisy trajectories containing 3 time courses—one for each variable. Then we formed 100 pairs of trajectories from the original and the conserved models, and 100 pairs from the original and the rewired models. Each pair is the input to *Q*-method/GSCA, and the output is a decision *p_d_*-value/divergence index evaluating strength of rewiring. It is somehow unexpected that GSCA performed well at a low noise level (SNR = 100 dB), suggesting comparison of chaotic systems is approachable. Nonetheless, the *Q*-method demonstrated an enormous advantage at both low and intermediate noise levels. Therefore, this example establishes the possibility to distinguish mechanistically changed chaotic systems.

### Prioritizing functional pathway rewiring among evolving yeasts

Although network rewiring is expected among related yeast species because they are mechanistically different biological systems, we hypothesize that the various functional pathways in them are not rewired to the same extent. We thus apply the *Q*-method to prioritize rewiring of 68 known KEGG pathways in yeast and reveal a consistent association between pathway interaction heterogeneity and TATA box disparity—a form of pathway rewiring. This thus offers a connection between mechanistic rewiring and observed divergent dynamics among the yeasts at a pathway scale.

Divergence in expression of single genes has been at least partially explained by the presence of TATA box in the promoter region of the genes^1^. TATA box is a short DNA sequence found in the promoter region of some genes and is highly conserved in eukaryotes. It is the binding site for general transcription factors or histones to facilitate or block transcription. Tirosh et al^1^ measured time-course transcriptomes of four yeast species (*S. cerevisiae*, *S. paradoxus*, *S. mikatae*, and *S. kudriavzevil*) in response to five environmental stresses including DNA damage, heat shock, oxidative stress, carbon source switch, and nitrogen starvation. Using this data set (Gene Expression Omnibus accession number GSE3406), we examined 68 metabolic and signaling pathways from KEGG^17^ and performed on each pathway 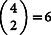 comparative runs for its rewiring across the four yeast species. The setup and complete result of this study are detailed in Supplementary Text 3.3.

The obtained *Q_d_*-statistics are normalized to 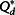 for comparability across pathways and also for regression analysis. The median pathway interaction heterogeneity score among the six pairs of species was used to summarize the overall heterogeneity of each pathway among the four species. They are summarized in Supplementary Table S1. Although the various metabolic pathways show wide range of median pathway heterogeneity, cell signaling and cell cycle pathways are among the most conserved pathways among the four yeast species. The large pathway heterogeneity variation observed among the pathways supports our hypothesis of unequal extent of pathway rewiring among the yeasts. This result can thus help us zoom into the most unique pathways that drive different phenotypes.

**Figure 5:**
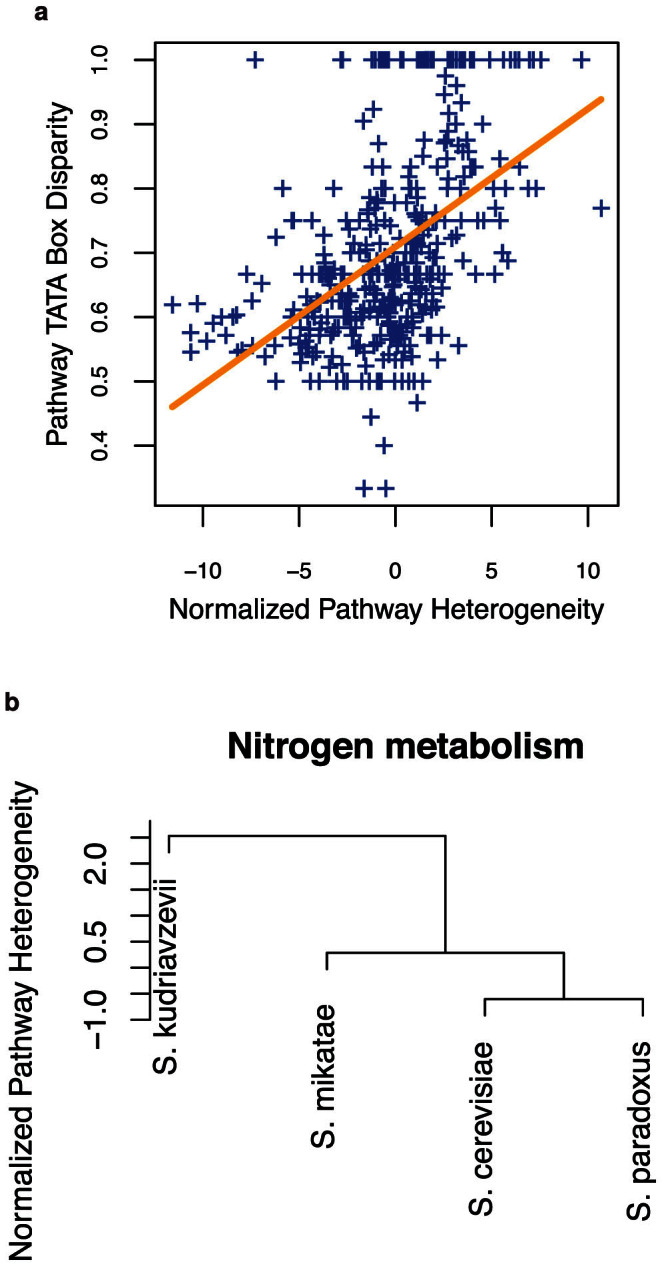
Pathway heterogeneity between transcriptomes predicts evolutionary relationships among four yeast species. The four yeast species are *S. kudriavzevii*, *S. cerevisiae*, *S. mikatae*, and *S. paradoxus*. (a) Normalized pathway heterogeneity is associated with TATA box disparity between genomes across four yeast species. Each point represents heterogeneity and TATA box disparity of one of 68 pathways between one of 6 possible pairs among 4 yeast species. (b) Clustering of the yeast species by normalized heterogeneity of the nitrogen metabolism pathway generates a tree consistent with the evolutionary relationship among the four yeast species. A qualitatively identical phylogenetic tree^22^ suggests that *S. kudriavzevii* diverged from *S. cerevisiae* within the last 20 million years, followed by the divergence of *S. mikatae*, and then the split of *S. paradoxus* within the last 10 million years.

We next examined whether TATA box disparity is a factor contributing to the observed pathway heterogeneity. We define pathway TATA box disparity between two species as the ratio of the number of genes with a TATA box in only one species to the number of genes with a TATA box in either species within a pathway. Normalized pathway heterogeneity and TATA box disparity, given in Supplementary Table S2 for all pathway pairs among the four species, exhibit a strong positive correlation at *P* = 1.09 × 10^−23^ (Fig. 5(a)), complement to the conclusion that the presence of TATA box in the promoter of a gene increases its expression variability^1^. For example, the nitrogen metabolism pathway showed a remarkably positive correlation between normalized pathway heterogeneity and pathway TATA box disparity. Nitrogen metabolism is necessary to reduce intracellular nitrogen toxicity in response to ammonium. The pathway varied in conservation between species in terms of gene expression and the conservation of TATA boxes. Between *S. paradoxus* and *S. cerevisiae* the normalized heterogeneity was low (−0.6) with low TATA box disparity (40%) yet between *S. paradoxus* and *S. kudriavzevii* the normalized heterogeneity was high (7.54) with 100% TATA box disparity. By hierarchical clustering, a tree is obtained based on the pathway heterogeneity between pairs of the four yeast species (Fig. 5(b)). *S. paradoxus* shared a common ancestor more recently in the last 10 million years with *S. cerevisiae* than it did with *S. kudriavzevii* in the last 20 million years^22^, which partially explains the differences in heterogeneity of this pathway. This pathway is biologically interesting as it has remained relatively conserved between some species yet has diverged greatly between others which demonstrates that although a pathway may seem conserved with low TATA box disparity, it is possible that between another species of the same genus that the same pathway can become highly rewired with evolutionary time. See discussion of additional pathways in Supplementary Text 3.3.4.

## Discussion

When the sample size of an experiment is small, linear or nonlinear correlation has been widely used to infer interactions in molecular networks for mathematical convenience and statistical efficiency. However, correlation is not the language for dynamics in biological systems. Instead, it is the control of the rate of change (derivative) of a target molecule by a regulator molecule that is most often characterized in established mathematical models of molecular interactions. Description of such control is typically given in the language of differential equations. In signal transduction models, the rate at which a protein is phosphorylated is mediated by the corresponding kinase^23^; in transcription kinetics models, binding-site occupancy by transcription factors is used to calculate the rate of change of target RNAs via differential equations^24^; in metabolic network models, reaction rates are again determined by enzyme concentration^25^. As the rate of change of molecule abundance in these models is not constant, the correlation coefficient between cause and effect variables changes with the concentration of the causative factors. This implies that differential correlation between variables in even two conserved systems is not always zero. Therefore, differential-correlation-based pathway analyses could flag conserved pathways as rewired or vice versa due to its overly simplistic treatment of system dynamics using only trajectories but not their derivatives.

We have thus demonstrated that *Q*-method can overcome the inadequacies of differential-correlation-based pathway rewiring detection approaches. The nearly zero AUC under the ROC curves of GSCA in some cases, e.g., BIOMD0000000005 (Fig. 2b,c,d) and BIOMD0000000067 (Supplementary Fig. S1 to S4) appeared to suggest a systematic bias which could have been corrected by reverse each pathway rewiring decision made by GSCA. However, doing so would make GSCA unfavorably score a nearly zero AUC in other cases, e.g., BIOMD0000000021 and BIOMD0000000035 (Supplementary Fig. S1, S2, S3). This large inconsistency of GSCA performance reveals the inherent flaws of the different-correlation paradigm when used to study dynamical systems. As GSCA examines linear dependencies among variables in observed trajectory patterns, it can make false positive decisions when the trajectory patterns are divergent simply as a consequence of different initial states of the same pathway; on the other hand, when two trajectory patterns from a rewired pathway are dynamically traversed differently but happen to share a similar shape and thus correlation coefficients, GSCA could make false negative decisions. *Q*-method overcomes all these issues by detecting potentially nonlinear patterns utilizing both the rate of change and the trajectory pattern.

The usefulness of *Q*-method depends on the availability of time course data and accurate estimation of the rate of change. Although not all experimental design offers such opportunities to study pathway rewiring, high-resolution temporal sampling technologies are being rapidly developed to study vaccine response^26^ or record spatiotemporal trajectories of molecules^27^. The requirement of a given super-pathway topology is not a limiting factor to the *Q*-method as the topology can be alternatively obtained using various network inference approaches. The BioModels testing data set used in our study covered a broad spectrum of biological processes including cell cycle, circadian clock, glycolysis, metabolic oscillation, and calcium oscillation. We also tested and compared methods on a chaotic Lotka-Volterra predator-prey system. The corresponding benchmark results supported the effectiveness of the *Q*-method. It is informative to point out that most examples we tested exhibited large dynamic ranges, a necessary condition to track how mechanistically-rewired pathways may differentially respond to external stimuli. When no such wide dynamic ranges are recorded with the observed data, it is possible that a rewiring decision could be unreliably made, by either the *Q*-method or other methods based on rate-of-change variations. Under sufficient data conditions, by discerning whether a pathway has been mechanistically rewired, the *Q*-method can eliminate many pathways with divergent responses only as a consequence of pathway input, instead of rewiring. In order to determine what biological variations may have caused the rewiring, one will need to investigate additional information regarding the rewired pathway. This can include sequences of the involved proteins or transcripts and their methylation profiles or histone modification data. For example, when both genome sequences and gene expression are provided for the species being compared for rewiring, we can associate TATA box sequence presence/absence for each gene with its calculated interaction heterogeneity based on expression levels. In this manner, we can establish a model where TATA box disparity is a model parameter to predict interaction heterogeneity or rewiring. This will eventually lead to testable hypotheses on the biological causes for rewiring. We thus evaluated how detected pathway rewiring as a phenotypical change could be indicative of the underlying genotypical modifications among four yeast species. Based on gene expression data demonstrating large dynamic ranges collected in these yeasts subjected to five stress conditions, we found a strong positive correlation between pathway interaction heterogeneity and TATA box disparity. This confirms the power of *Q*-method to discover pathway rewiring indeed explainable by genotypical variations in biological systems.

## Conclusions

The *Q*-method has addressed three challenges called forth by Khatri et al^12^, by explicitly modeling pathway dynamics, distinguishing mechanistic versus superficial changes, and requiring an inclusive but not necessarily exact pathway topology as input. Simulation studies on diverse types of model, including chaotic systems, suggest the effectiveness and generality of the *Q*-method. Most importantly, the *Q*-method predicted rewired pathways by their interaction heterogeneity across yeast species. The correlation between pathway heterogeneity and TATA box disparity suggests a potential effect-cause relationship linked by the inferred pathway rewiring. The *Q*-method is readily extendable to multiple conditions with the number of heterogeneous models equal to the number of conditions. The principle that we have established to compare ODE models is highly innovative. Although we have illustrated the *Q*-method using an additive nonlinear DSM, the same principle is immediately generalizable to other forms of ODE models. As time course experimental design, genome sequences, and epigenome markers become increasingly available, we expect that the *Q*-method will link observed changed dynamics to various causative genetic and even epigenetic factors at the pathway level pointing to evolved biological functions.

## Supplementary Material

Supplementary Information Supplementary Information

## Figures and Tables

**Table 1:** Setup of BioModels simulation studies for each model. These values are used to perturb original models from the BioModels database^13^ to generate conserved and differential pathway models

Model name	Mean	Std	Upper bound	Lower bound	Start time	End time	Time step
	*μ*	*σ*	*U*	*L*	*t*_0_	*t*_100_	Δ*t*
BIOMD0000000005: Interactions of cdc2 and cyclin in cell devision cycle^21^	3	0.1	0.005	0.001	0	100	1
BIOMD0000000008: Cell division cycle dynamics^28^	2	0.1	0.5	0.1	0	100	1
BIOMD0000000021: Circadian oscillations of PER & TIM proteins in *Drosophila*^29^	2	0.1	0.009	0.005	0	20	0.2
BIOMD0000000035: Circadian oscillations^30^	3	0.1	0.5	0.1	0	40	0.4
BIOMD0000000042: Oscillations in glycolysis^31^	1.8	0.1	0.05	0.03	0	100	1
BIOMD0000000043: Intracellular calcium oscillations^32^	1.095	0.001	0.04	0.02	0	10	0.1
BIOMD0000000045: Intracellular calcium oscillations^32^	1.9	0.01	0.05	0.01	0	30	0.3
BIOMD0000000061: Glycolysis in *S. cerevisiae*^33^	1.1	0.01	0.005	0.001	0	30	0.3
BIOMD0000000067: Synthetic gene-metabolic oscillator^34^	3	0.01	0.009	0.001	0	100	1
BIOMD0000000069: Activation of Src at mitosis^35^	2	0.1	0.05	0.01	0	50	0.5
BIOMD0000000073: Mammalian circadian clock^36^	2	0.1	0.09	0.05	0	300	3
BIOMD0000000078: Mammalian circadian clock^36^	2	0.1	0.9	0.1	0	400	4

To create a differential model, each parameter in the original model was multiplied by a random number normally distributed with mean *μ* and standard deviation *σ*. The initial value of a variable was changed by adding a random number uniformly distributed in [*L*, *U*] to the original initial state. Given a model and its initial state, we sampled time points at *t*_0_ to *t*_100_, uniformly spaced with a model-dependent time step Δ*t*.

**Table 2:** Runtime of BioModels simulation studies for each model. Runtime for each BioModels model^13^ includes simulation and comparison of 100 pairs of conserved models, and 100 pairs of differential models, based on the same original model

Model name	Number of molecular species	Runtime (seconds)
BIOMD0000000005	9	539
BIOMD0000000008	5	176
BIOMD0000000021	10	341
BIOMD0000000035	10	2194
BIOMD0000000042	15	1567
BIOMD0000000043	5	209
BIOMD0000000045	4	396
BIOMD0000000061	25	4260
BIOMD0000000067	8	194
BIOMD0000000069	10	248
BIOMD0000000073	16	740
BIOMD0000000078	16	811

The simulation program was implemented in R and the Q-method was implemented in a mixture of R and C++ programming languages. The runtime also includes GSCA implemented in R. The runtime was obtained on an iMac with 2.93 GHz Intel Core i7 16 GB 1333 MHz DDR3 memory running OS X 10.10.1.

**Table 3:** The *Q*-method gains an overwhelming advantage over GSCA in average area under the ROC curves and their standard deviations

	Average area under the ROC curve			
	SNR = 100 dB	20 dB	10 dB	0 dB
***Q*-method**	0.98 ± 0.027	0.90 ± 0.15	0.79 ± 0.17	0.63 ± 0.11
**GSCA**	0.49 ± 0.38	0.52 ± 0.35	0.55 ± 0.30	0.53 ± 0.17

The results were obtained on 12 BioModels at four different signal-to-noise ratios (SNRs). A larger area under the curve (AUC) indicates a better performance.
