# Supplementary material for: Discerning mechanistically rewired biological pathways by cumulative interaction heterogeneity statistics
Source: Sci Rep. 2015 Apr 28;5:9634. doi: 10.1038/srep09634 (PMC4894439; doi:10.1038/srep09634)
Supplement: Supplementary Information [file srep09634-s1.pdf]

SUPPLEMENTARY INFORMATION

Discerning mechanistically rewired biological pathways by cumulative interaction heterogeneity statistics

Travis B. Cotton [1,3], Hien H. Nguyen [1], Joseph I. Said [2], Zhengyu Ouyang [1,4], Jinfa Zhang [2] and Mingzhou Song [1,*]

[1] Department of Computer Science, New Mexico State University, Las Cruces, New Mexico, USA
[2] Department of Plant and Environmental Sciences, New Mexico State University, Las Cruces, New Mexico, USA

Current addresses:
[3] High Performance Computing Center, Texas Tech University, Lubbock, Texas, USA
[4] Ragon Institute of Massachusetts General Hospital, MIT and Harvard University, Cambridge, Massachusetts, USA

* To whom correspondence may be addressed: joemsong@cs.nmsu.edu

Contents

1 Supplementary Figures **1**

2 Supplementary Tables **6**

3 Supplementary Text **19**
3.1 Dynamical system model reconstruction 19
3.2 Comparable performance of GSCA and the Q-method at various noise levels 19
3.3 Predicting rewired pathways among four yeast species 20
3.3.1 TATA box detection 21
3.3.2 Pathway TATA box disparity 21
3.3.3 Normalized pathway interaction heterogeneity 22
3.3.4 Pathway interaction heterogeneity is consistent with TATA box disparity among yeast species 22

1 Supplementary Figures

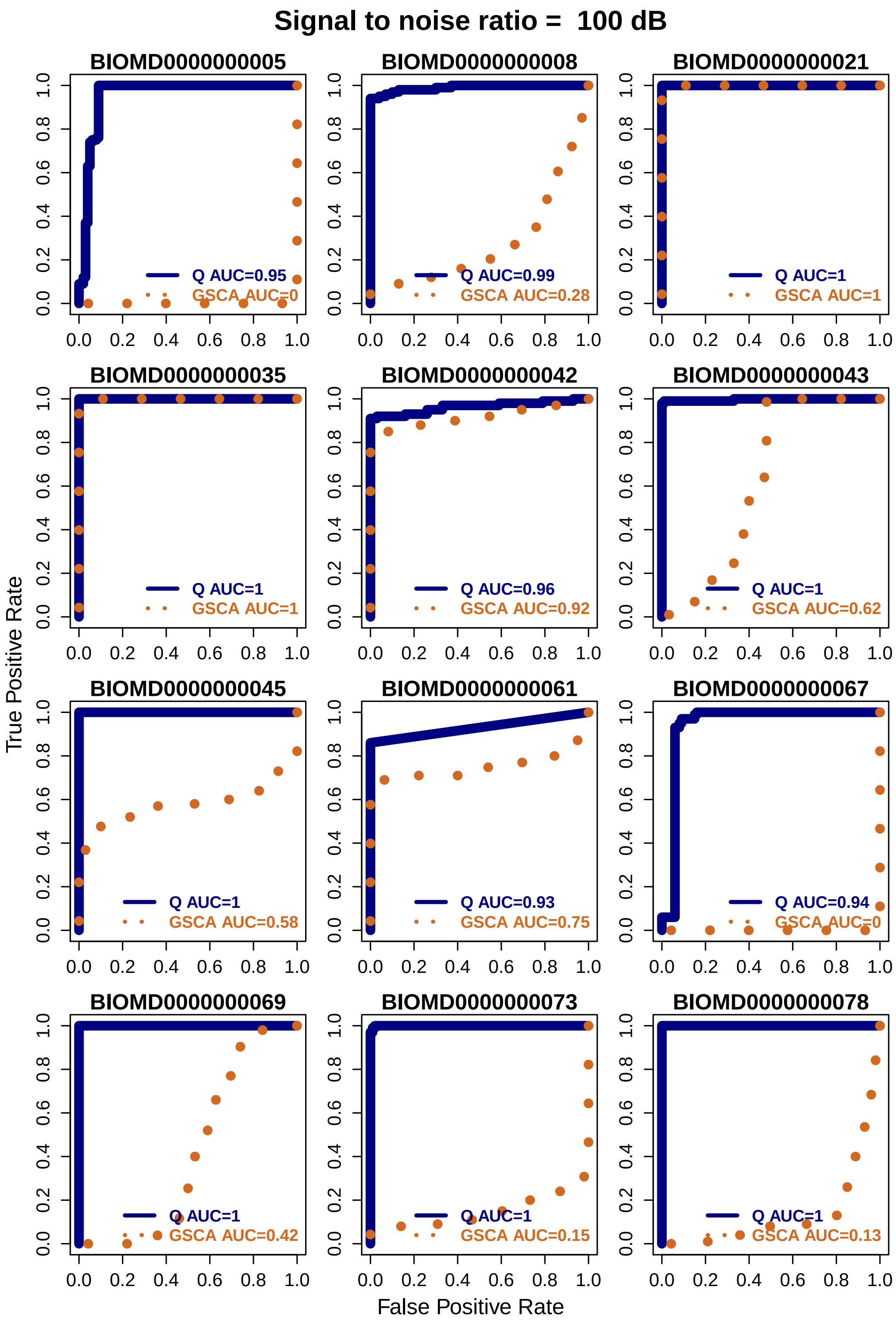

Figure S1. The ROC curves of the *Q*-method (blue) and Gene Set Correlation Analysis (GSCA) (orange) at a signal-to-noise ratio (SNR) of 100 dB. At this level the *Q*-method is almost perfect but GSCA performed far worse except in three models. The results were obtained on data at 100 dB from 12 BioModels (Chelliah et al., 2013). Each plot summarizes 100 conserved and 100 differential model pairs based on one original model among the 12 BioModels. AUC in the legends stands for area under ROC curve. A larger AUC indicates a better performance.

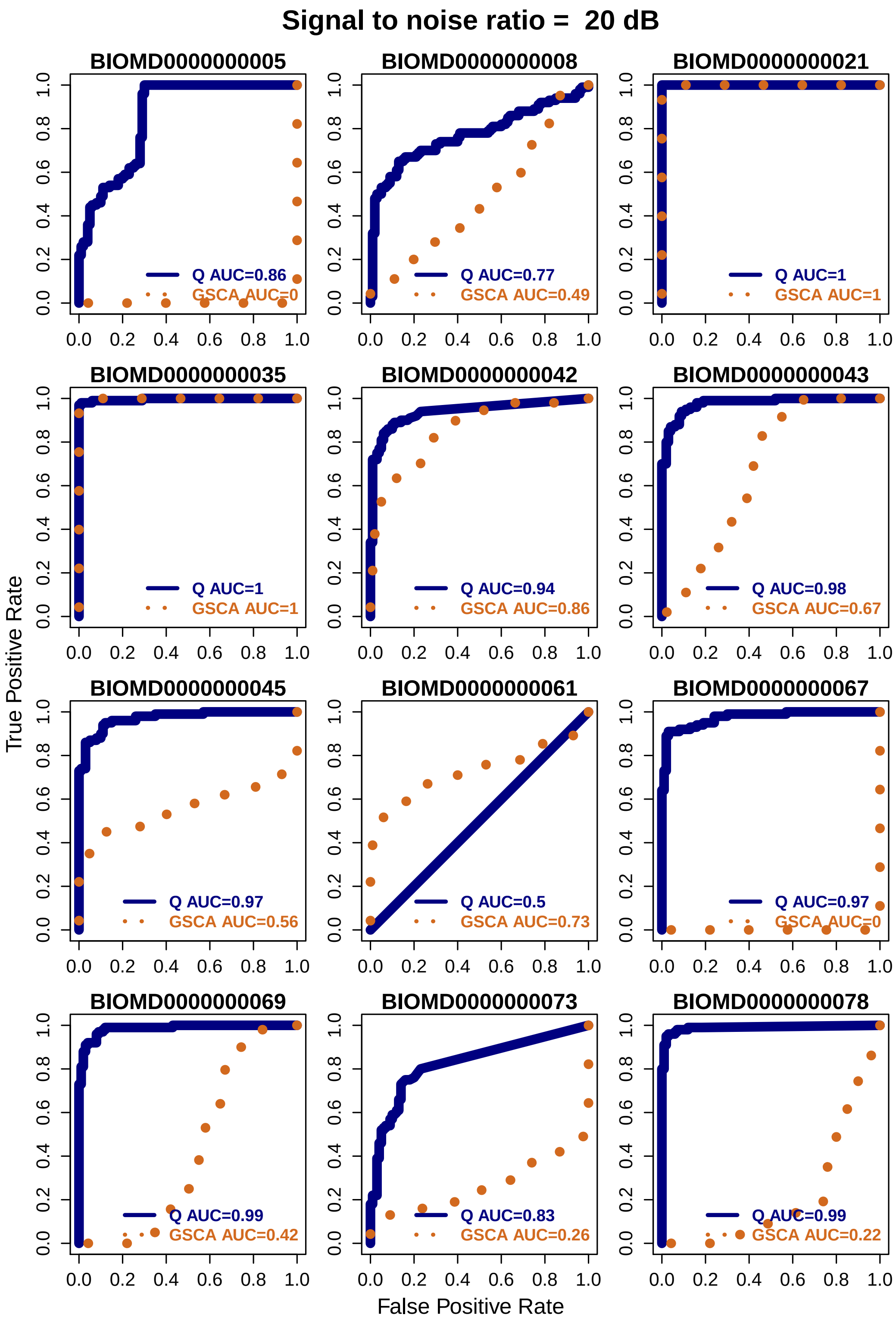

Figure S2. The ROC curves of the Q-method (blue) and GSCA (orange) at a signal-to-noise ratio of 20 dB. At this level the Q-method outperformed GSCA considerably on 8 models and was equal or slightly better in 4 models.

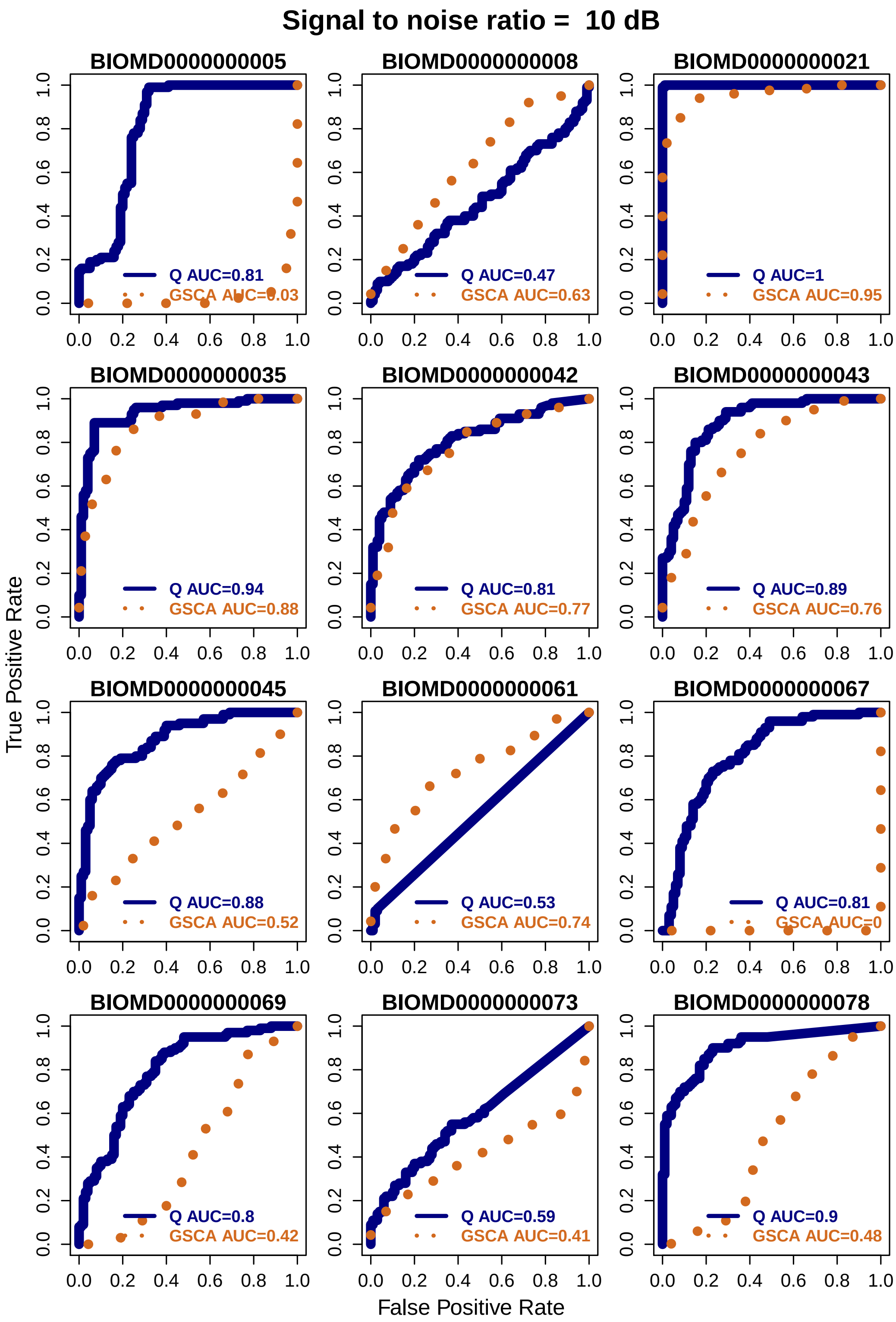

Figure S3. The ROC curves of the *Q*-method (blue) and GSCA (orange) at a signal-to-noise ratio of 10 dB. At this level the *Q*-method outperformed GSCA on 9 models and slightly underperformed in 3 models.

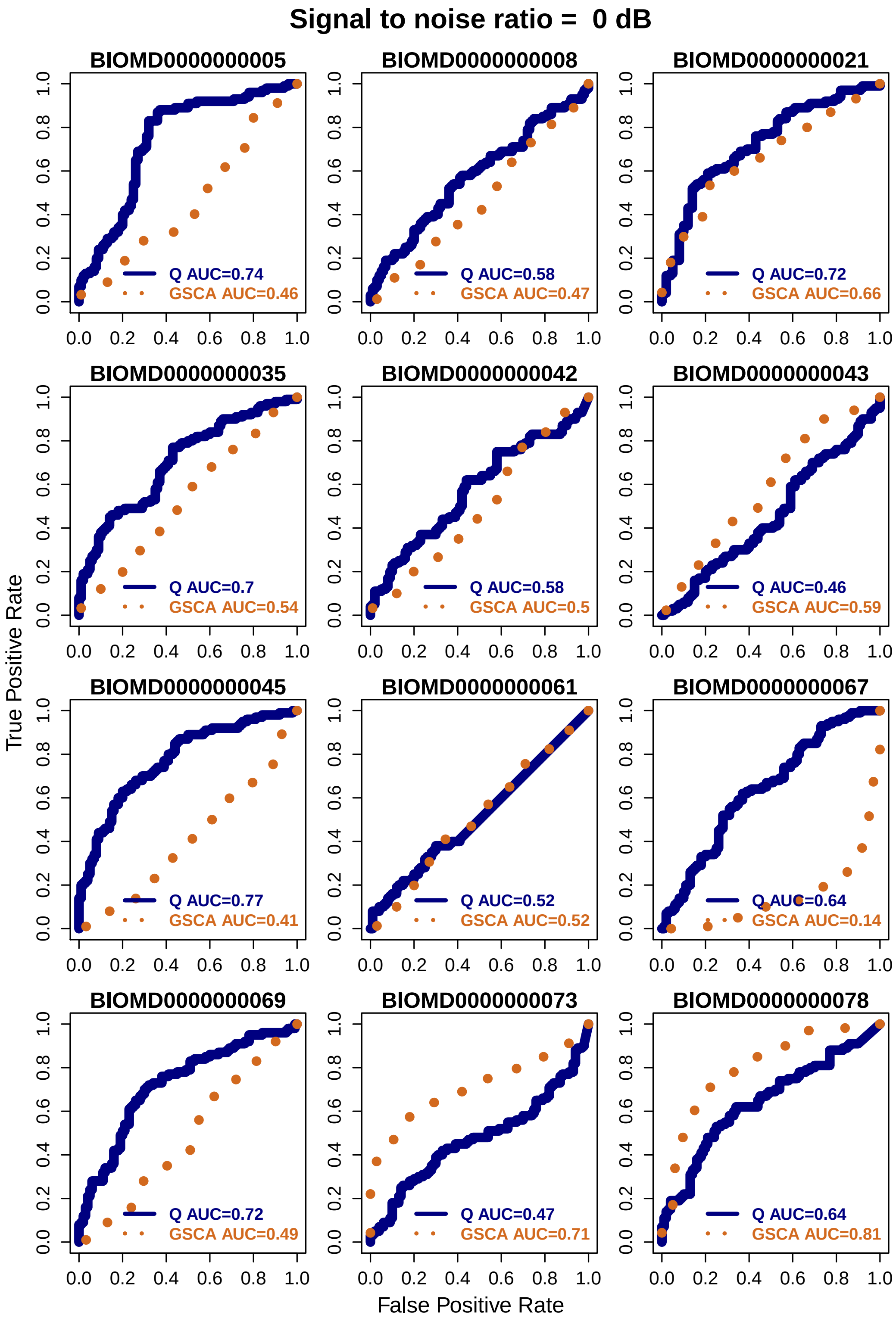

Figure S4. The ROC curves of the *Q*-method (blue) and GSCA (orange) at a signal-to-noise ratio of 0 dB. At this level the *Q*-method outperformed GSCA on 8 models and underperformed in 4 models.

2 Supplementary Tables

Table S1. Prioritized pathways ranked by median heterogeneity among the four yeast species. The pathway heterogeneity Q_d^* is the median taken over the six pairs among the four yeast species from Table S4.

Pathway ID	Pathway Name	Median normalized pathway heterogeneity Q_d^* among four yeast species
sce00513	Various types of N-glycan biosynthesis	5.57
sce00785	Lipoic acid metabolism	4.98
sce00350	Tyrosine metabolism	3.76
sce00360	Phenylalanine metabolism	3.14
sce00910	Nitrogen metabolism	2.52
sce04144	Endocytosis	2.45
sce00790	Folate biosynthesis	2.36
sce00920	Sulfur metabolism	2.19
sce00562	Inositol phosphate metabolism	1.98
sce00290	Valine, leucine and isoleucine biosynthesis	1.93
sce00750	Vitamin B6 metabolism	1.76
sce04145	Phagosome	1.72
sce04122	Sulfur relay system	1.66
sce00650	Butanoate metabolism	1.60
sce00561	Glycerolipid metabolism	1.59
sce00970	Aminoacyl-tRNA biosynthesis	1.57
sce00909	Sesquiterpenoid biosynthesis	1.45
sce04130	SNARE interactions in vesicular transport	1.40
sce00450	Selenocompound metabolism	0.96
sce00500	Starch and sucrose metabolism	0.93
sce00071	Fatty acid metabolism	0.88
sce00780	Biotin metabolism	0.87
sce00280	Valine, leucine and isoleucine degradation	0.86
sce00061	Fatty acid biosynthesis	0.74
sce00565	Ether lipid metabolism	0.69
sce00340	Histidine metabolism	0.67
sce00630	Glyoxylate and dicarboxylate metabolism	0.53
sce00062	Fatty acid elongation in mitochondria	0.50
sce03018	RNA degradation	0.46
sce04070	Phosphatidylinositol signaling system	0.16
sce00040	Pentose and glucuronate interconversions	0.06
sce03015	mRNA surveillance pathway	-0.21
sce00300	Lysine biosynthesis	-0.27
sce00510	N-Glycan biosynthesis	-0.27
sce00730	Thiamine metabolism	-0.33
sce04141	Protein processing in endoplasmic reticulum	-0.56
sce00100	Steroid biosynthesis	-0.56
sce00410	beta-Alanine metabolism	-0.61
sce00380	Tryptophan metabolism	-0.75
sce00900	Terpenoid backbone biosynthesis	-0.83
sce00051	Fructose and mannose metabolism	-1.02
sce00600	Sphingolipid metabolism	-1.30
sce00563	Glycosylphosphatidylinositol(GPI)-anchor biosynthesis	-1.34
sce03013	RNA transport	-1.37
sce00860	Porphyrin and chlorophyll metabolism	-1.43
sce00520	Amino sugar and nucleotide sugar metabolism	-1.80
sce00400	Phenylalanine, tyrosine and tryptophan biosynthesis	-1.93

sce00670	One carbon pool by folate	-2.25
sce00680	Methane metabolism	-2.38
sce00330	Arginine and proline metabolism	-2.38
sce00620	Pyruvate metabolism	-2.45
sce00564	Glycerophospholipid metabolism	-2.50
sce00480	Glutathione metabolism	-2.51
sce00770	Pantothenate and CoA biosynthesis	-2.60
sce00250	Alanine, aspartate and glutamate metabolism	-2.61
sce00760	Nicotinate and nicotinamide metabolism	-2.63
sce00740	Riboflavin metabolism	-2.74
sce00260	Glycine, serine and threonine metabolism	-2.74
sce04011	MAPK signaling pathway - yeast	-2.82
sce00030	Pentose phosphate pathway	-4.20
sce00052	Galactose metabolism	-4.32
sce03008	Ribosome biogenesis in eukaryotes	-4.53
sce00010	Glycolysis / Gluconeogenesis	-6.44
sce00020	Citrate cycle (TCA cycle)	-6.53
sce00270	Cysteine and methionine metabolism	-6.58
sce00240	Pyrimidine metabolism	-7.98
sce04113	Meiosis - yeast	-10.30
sce04111	Cell cycle - yeast	-10.63

Table S2. Pathway heterogeneity across yeast species. The pathway heterogeneity Q_d^* is normalized from Q_d-statistics. The pathway TATA box disparity is calculated as the ratio of the number of TATA-containing genes in only one species (in 2 compared species) and the number of TATA-containing genes in either one of 2 compared species.

Pathway ID	Pathway Name	Species 1	Species 2	Normalized pathway heterogeneity Q_d^*	Pathway TATA box disparity
sce00513	Various types of N-glycan biosynthesis	S. cerevisiae	S. paradoxus	10.68	0.77
sce00790	Folate biosynthesis	S. cerevisiae	S. paradoxus	9.65	1.00
sce00910	Nitrogen metabolism	S. paradoxus	S. kudriavzevii	7.54	1.00
sce00630	Glyoxylate and dicarboxylate metabolism	S. paradoxus	S. kudriavzevii	7.15	1.00
sce00513	Various types of N-glycan biosynthesis	S. paradoxus	S. mikatae	7.29	0.80
sce00790	Folate biosynthesis	S. cerevisiae	S. mikatae	6.95	1.00
sce00350	Tyrosine metabolism	S. paradoxus	S. kudriavzevii	6.89	0.80
sce00051	Fructose and mannose metabolism	S. paradoxus	S. kudriavzevii	6.21	1.00
sce00910	Nitrogen metabolism	S. mikatae	S. kudriavzevii	6.39	1.00
sce00620	Pyruvate metabolism	S. paradoxus	S. kudriavzevii	5.82	0.69
sce00785	Lipoic acid metabolism	S. cerevisiae	S. paradoxus	6.43	0.83
sce00270	Cysteine and methionine metabolism	S. paradoxus	S. mikatae	5.42	0.75
sce00513	Various types of N-glycan biosynthesis	S. paradoxus	S. kudriavzevii	5.96	1.00
sce00562	Inositol phosphate metabolism	S. paradoxus	S. kudriavzevii	5.60	1.00
sce00785	Lipoic acid metabolism	S. cerevisiae	S. kudriavzevii	5.70	0.80
sce00562	Inositol phosphate metabolism	S. cerevisiae	S. kudriavzevii	5.41	0.85
sce00500	Starch and sucrose metabolism	S. paradoxus	S. kudriavzevii	5.14	0.67
sce00350	Tyrosine metabolism	S. mikatae	S. kudriavzevii	5.55	0.70
sce00062	Fatty acid elongation in mitochondria	S. cerevisiae	S. paradoxus	5.53	0.70
sce00513	Various types of N-glycan biosynthesis	S. cerevisiae	S. mikatae	5.19	0.77
sce00785	Lipoic acid metabolism	S. cerevisiae	S. mikatae	5.08	0.80
sce00785	Lipoic acid metabolism	S. paradoxus	S. kudriavzevii	4.88	1.00
sce00562	Inositol phosphate metabolism	S. mikatae	S. kudriavzevii	4.51	0.90
sce00513	Various types of N-glycan biosynthesis	S. cerevisiae	S. kudriavzevii	4.57	0.75
sce00290	Valine, leucine and isoleucine biosynthesis	S. mikatae	S. kudriavzevii	4.25	0.75
sce00510	N-Glycan biosynthesis	S. paradoxus	S. kudriavzevii	4.04	1.00
sce00400	Phenylalanine, tyrosine and tryptophan biosynthesis	S. cerevisiae	S. paradoxus	3.90	0.83
sce00350	Tyrosine metabolism	S. cerevisiae	S. kudriavzevii	4.11	0.83
sce00513	Various types of N-glycan biosynthesis	S. mikatae	S. kudriavzevii	3.88	1.00
sce00360	Phenylalanine metabolism	S. paradoxus	S. kudriavzevii	4.01	0.83
sce04145	Phagosome	S. cerevisiae	S. paradoxus	4.15	0.67
sce00051	Fructose and mannose metabolism	S. mikatae	S. kudriavzevii	3.56	1.00
sce00340	Histidine metabolism	S. cerevisiae	S. paradoxus	3.69	0.88
sce00670	One carbon pool by folate	S. paradoxus	S. kudriavzevii	3.56	1.00
sce00565	Ether lipid metabolism	S. paradoxus	S. mikatae	4.02	1.00
sce04122	Sulfur relay system	S. paradoxus	S. mikatae	3.77	0.86
sce00630	Glyoxylate and dicarboxylate metabolism	S. cerevisiae	S. kudriavzevii	3.40	0.75
sce00730	Thiamine metabolism	S. cerevisiae	S. mikatae	3.50	0.69
sce00600	Sphingolipid metabolism	S. cerevisiae	S. paradoxus	3.49	0.86
sce00970	Aminoacyl-tRNA biosynthesis	S. paradoxus	S. mikatae	3.58	1.00
sce00790	Folate biosynthesis	S. paradoxus	S. mikatae	3.46	1.00
sce00360	Phenylalanine metabolism	S. cerevisiae	S. paradoxus	3.44	1.00
sce00290	Valine, leucine and isoleucine biosynthesis	S. paradoxus	S. kudriavzevii	3.34	1.00
sce00970	Aminoacyl-tRNA biosynthesis	S. cerevisiae	S. paradoxus	3.37	0.80
sce00360	Phenylalanine metabolism	S. cerevisiae	S. mikatae	3.36	1.00

sce00750	Vitamin B6 metabolism	S. cerevisiae	S. kudriavzevii	3.27	0.56
sce04144	Endocytosis	S. paradoxus	S. kudriavzevii	3.39	1.00
sce00410	beta-Alanine metabolism	S. paradoxus	S. mikatae	3.16	0.96
sce04122	Sulfur relay system	S. cerevisiae	S. paradoxus	3.55	0.82
sce00350	Tyrosine metabolism	S. paradoxus	S. mikatae	3.41	0.93
sce00040	Pentose and glucuronate interconversions	S. cerevisiae	S. paradoxus	3.25	0.87
sce00561	Glycerolipid metabolism	S. cerevisiae	S. paradoxus	3.13	0.90
sce00780	Biotin metabolism	S. paradoxus	S. mikatae	3.16	0.73
sce00970	Aminoacyl-tRNA biosynthesis	S. paradoxus	S. kudriavzevii	3.16	1.00
sce00920	Sulfur metabolism	S. cerevisiae	S. kudriavzevii	2.97	1.00
sce04130	SNARE interactions in vesicular transport	S. paradoxus	S. kudriavzevii	2.86	1.00
sce00360	Phenylalanine metabolism	S. mikatae	S. kudriavzevii	2.92	0.72
sce00561	Glycerolipid metabolism	S. cerevisiae	S. kudriavzevii	2.72	1.00
sce04070	Phosphatidylinositol signaling system	S. cerevisiae	S. paradoxus	2.66	0.85
sce04144	Endocytosis	S. mikatae	S. kudriavzevii	2.75	0.92
sce00280	Valine, leucine and isoleucine degradation	S. cerevisiae	S. kudriavzevii	2.73	0.81
sce04144	Endocytosis	S. cerevisiae	S. kudriavzevii	2.69	0.80
sce00350	Tyrosine metabolism	S. cerevisiae	S. paradoxus	2.71	0.89
sce00290	Valine, leucine and isoleucine biosynthesis	S. cerevisiae	S. kudriavzevii	2.55	0.75
sce00360	Phenylalanine metabolism	S. cerevisiae	S. kudriavzevii	2.64	0.88
sce04141	Protein processing in endoplasmic reticulum	S. cerevisiae	S. paradoxus	2.53	0.87
sce00910	Nitrogen metabolism	S. cerevisiae	S. kudriavzevii	2.53	0.95
sce00785	Lipoic acid metabolism	S. paradoxus	S. mikatae	2.58	0.98
sce00910	Nitrogen metabolism	S. cerevisiae	S. mikatae	2.52	0.67
sce04130	SNARE interactions in vesicular transport	S. cerevisiae	S. paradoxus	2.41	0.65
sce00500	Starch and sucrose metabolism	S. mikatae	S. kudriavzevii	2.24	0.67
sce00450	Selenocompound metabolism	S. cerevisiae	S. paradoxus	2.38	0.57
sce00920	Sulfur metabolism	S. cerevisiae	S. paradoxus	2.22	0.67
sce00520	Amino sugar and nucleotide sugar metabolism	S. cerevisiae	S. paradoxus	2.16	0.83
sce00920	Sulfur metabolism	S. cerevisiae	S. mikatae	2.16	0.71
sce00650	Butanoate metabolism	S. cerevisiae	S. kudriavzevii	2.17	0.53
sce00750	Vitamin B6 metabolism	S. cerevisiae	S. mikatae	2.11	0.62
sce00750	Vitamin B6 metabolism	S. cerevisiae	S. paradoxus	2.10	0.69
sce04145	Phagosome	S. paradoxus	S. mikatae	2.07	0.76
sce00500	Starch and sucrose metabolism	S. cerevisiae	S. kudriavzevii	1.91	0.67
sce00260	Glycine, serine and threonine metabolism	S. cerevisiae	S. paradoxus	1.95	1.00
sce00909	Sesquiterpenoid biosynthesis	S. cerevisiae	S. mikatae	2.10	0.75
sce00770	Pantothenate and CoA biosynthesis	S. paradoxus	S. mikatae	1.95	0.69
sce00260	Glycine, serine and threonine metabolism	S. cerevisiae	S. mikatae	1.91	0.64
sce00340	Histidine metabolism	S. paradoxus	S. kudriavzevii	1.93	0.75
sce00650	Butanoate metabolism	S. cerevisiae	S. paradoxus	1.93	0.63
sce03015	mRNA surveillance pathway	S. paradoxus	S. kudriavzevii	1.93	1.00
sce00561	Glycerolipid metabolism	S. cerevisiae	S. mikatae	1.86	0.61
sce00650	Butanoate metabolism	S. mikatae	S. kudriavzevii	1.84	1.00
sce00785	Lipoic acid metabolism	S. mikatae	S. kudriavzevii	1.80	0.67
sce00071	Fatty acid metabolism	S. cerevisiae	S. paradoxus	1.81	0.57
sce04145	Phagosome	S. cerevisiae	S. mikatae	1.79	0.66
sce00909	Sesquiterpenoid biosynthesis	S. cerevisiae	S. paradoxus	1.79	0.57
sce00300	Lysine biosynthesis	S. paradoxus	S. mikatae	1.69	0.74
sce04122	Sulfur relay system	S. cerevisiae	S. kudriavzevii	1.75	0.70
sce00730	Thiamine metabolism	S. cerevisiae	S. paradoxus	1.67	1.00
sce04145	Phagosome	S. mikatae	S. kudriavzevii	1.66	0.57
sce04130	SNARE interactions in vesicular transport	S. paradoxus	S. mikatae	1.62	0.67
sce00760	Nicotinate and nicotinamide metabolism	S. cerevisiae	S. paradoxus	1.55	0.62
sce04122	Sulfur relay system	S. paradoxus	S. kudriavzevii	1.57	0.75
sce00909	Sesquiterpenoid biosynthesis	S. cerevisiae	S. kudriavzevii	1.55	1.00

sce00565	Ether lipid metabolism	S. cerevisiae	S. mikatae	1.57	0.64
sce00510	N-Glycan biosynthesis	S. cerevisiae	S. paradoxus	1.50	1.00
sce00280	Valine, leucine and isoleucine degradation	S. paradoxus	S. mikatae	1.48	0.88
sce00750	Vitamin B6 metabolism	S. paradoxus	S. kudriavzevii	1.43	0.73
sce00780	Biotin metabolism	S. cerevisiae	S. mikatae	1.41	1.00
sce00780	Biotin metabolism	S. cerevisiae	S. paradoxus	1.40	0.50
sce03015	mRNA surveillance pathway	S. cerevisiae	S. paradoxus	1.39	0.61
sce04122	Sulfur relay system	S. cerevisiae	S. mikatae	1.41	0.85
sce00650	Butanoate metabolism	S. paradoxus	S. kudriavzevii	1.37	0.76
sce00909	Sesquiterpenoid biosynthesis	S. mikatae	S. kudriavzevii	1.35	0.75
sce00561	Glycerolipid metabolism	S. paradoxus	S. kudriavzevii	1.33	0.71
sce00290	Valine, leucine and isoleucine biosynthesis	S. cerevisiae	S. paradoxus	1.32	1.00
sce04144	Endocytosis	S. cerevisiae	S. mikatae	1.32	0.64
sce00790	Folate biosynthesis	S. cerevisiae	S. kudriavzevii	1.25	0.67
sce00071	Fatty acid metabolism	S. paradoxus	S. mikatae	1.23	0.62
sce00630	Glyoxylate and dicarboxylate metabolism	S. mikatae	S. kudriavzevii	1.22	0.75
sce00061	Fatty acid biosynthesis	S. cerevisiae	S. kudriavzevii	1.22	0.64
sce04130	SNARE interactions in vesicular transport	S. mikatae	S. kudriavzevii	1.19	1.00
sce00450	Selenocompound metabolism	S. cerevisiae	S. mikatae	1.13	0.79
sce00450	Selenocompound metabolism	S. paradoxus	S. mikatae	1.12	0.71
sce00565	Ether lipid metabolism	S. cerevisiae	S. paradoxus	1.13	1.00
sce00563	Glycosylphosphatidylinositol(GPI)-anchor biosynthesis	S. paradoxus	S. kudriavzevii	1.11	0.47
sce00071	Fatty acid metabolism	S. paradoxus	S. kudriavzevii	1.09	0.67
sce00280	Valine, leucine and isoleucine degradation	S. cerevisiae	S. mikatae	1.08	0.80
sce00620	Pyruvate metabolism	S. mikatae	S. kudriavzevii	1.08	0.68
sce04145	Phagosome	S. paradoxus	S. kudriavzevii	1.07	0.76
sce00750	Vitamin B6 metabolism	S. paradoxus	S. mikatae	1.04	0.65
sce00350	Tyrosine metabolism	S. cerevisiae	S. mikatae	1.00	0.72
sce00380	Tryptophan metabolism	S. paradoxus	S. kudriavzevii	1.01	0.65
sce00564	Glycerophospholipid metabolism	S. paradoxus	S. kudriavzevii	1.00	0.76
sce00062	Fatty acid elongation in mitochondria	S. paradoxus	S. mikatae	0.99	0.72
sce00860	Porphyrin and chlorophyll metabolism	S. paradoxus	S. kudriavzevii	0.98	0.67
sce00062	Fatty acid elongation in mitochondria	S. paradoxus	S. kudriavzevii	0.97	0.67
sce03018	RNA degradation	S. cerevisiae	S. paradoxus	0.96	0.71
sce00680	Methane metabolism	S. paradoxus	S. mikatae	0.95	0.50
sce00909	Sesquiterpenoid biosynthesis	S. paradoxus	S. kudriavzevii	0.90	0.70
sce00061	Fatty acid biosynthesis	S. cerevisiae	S. mikatae	0.89	0.59
sce00360	Phenylalanine metabolism	S. paradoxus	S. mikatae	0.88	0.58
sce03018	RNA degradation	S. paradoxus	S. mikatae	0.84	0.54
sce00563	Glycosylphosphatidylinositol(GPI)-anchor biosynthesis	S. mikatae	S. kudriavzevii	0.85	0.60
sce00340	Histidine metabolism	S. cerevisiae	S. kudriavzevii	0.82	0.64
sce00300	Lysine biosynthesis	S. cerevisiae	S. paradoxus	0.79	0.70
sce04130	SNARE interactions in vesicular transport	S. cerevisiae	S. mikatae	0.79	0.80
sce00450	Selenocompound metabolism	S. paradoxus	S. kudriavzevii	0.80	0.63
sce00061	Fatty acid biosynthesis	S. paradoxus	S. kudriavzevii	0.77	0.83
sce00909	Sesquiterpenoid biosynthesis	S. paradoxus	S. mikatae	0.73	0.56
sce00100	Steroid biosynthesis	S. paradoxus	S. mikatae	0.70	0.80
sce00061	Fatty acid biosynthesis	S. cerevisiae	S. paradoxus	0.70	0.63
sce00071	Fatty acid metabolism	S. cerevisiae	S. kudriavzevii	0.68	0.82
sce00330	Arginine and proline metabolism	S. cerevisiae	S. paradoxus	0.68	0.50
sce00760	Nicotinate and nicotinamide metabolism	S. paradoxus	S. kudriavzevii	0.65	0.67
sce04070	Phosphatidylinositol signaling system	S. cerevisiae	S. mikatae	0.60	0.60
sce00290	Valine, leucine and isoleucine biosynthesis	S. cerevisiae	S. mikatae	0.59	0.60
sce00280	Valine, leucine and isoleucine degradation	S. mikatae	S. kudriavzevii	0.56	0.78
sce03018	RNA degradation	S. paradoxus	S. kudriavzevii	0.57	0.67

sce00410	beta-Alanine metabolism	S. cerevisiae	S. mikatae	0.58	0.59
sce00340	Histidine metabolism	S. mikatae	S. kudriavzevii	0.52	0.70
sce00061	Fatty acid biosynthesis	S. paradoxus	S. mikatae	0.49	0.67
sce00040	Pentose and glucuronate interconversions	S. paradoxus	S. mikatae	0.46	0.56
sce00100	Steroid biosynthesis	S. mikatae	S. kudriavzevii	0.44	0.68
sce00900	Terpenoid backbone biosynthesis	S. paradoxus	S. kudriavzevii	0.45	0.55
sce04070	Phosphatidylinositol signaling system	S. paradoxus	S. mikatae	0.38	1.00
sce03018	RNA degradation	S. cerevisiae	S. mikatae	0.36	0.58
sce00780	Biotin metabolism	S. mikatae	S. kudriavzevii	0.33	0.50
sce00040	Pentose and glucuronate interconversions	S. cerevisiae	S. mikatae	0.33	0.53
sce00780	Biotin metabolism	S. paradoxus	S. kudriavzevii	0.30	0.62
sce00910	Nitrogen metabolism	S. paradoxus	S. mikatae	0.29	0.57
sce00565	Ether lipid metabolism	S. paradoxus	S. kudriavzevii	0.26	0.59
sce00561	Glycerolipid metabolism	S. mikatae	S. kudriavzevii	0.26	1.00
sce00280	Valine, leucine and isoleucine degradation	S. paradoxus	S. kudriavzevii	0.22	0.72
sce00561	Glycerolipid metabolism	S. paradoxus	S. mikatae	0.23	0.59
sce04141	Protein processing in endoplasmic reticulum	S. cerevisiae	S. mikatae	0.20	0.57
sce00051	Fructose and mannose metabolism	S. cerevisiae	S. paradoxus	0.20	0.67
sce04145	Phagosome	S. cerevisiae	S. kudriavzevii	0.10	0.56
sce00650	Butanoate metabolism	S. cerevisiae	S. mikatae	0.12	0.67
sce00061	Fatty acid biosynthesis	S. mikatae	S. kudriavzevii	0.11	0.57
sce00290	Valine, leucine and isoleucine biosynthesis	S. paradoxus	S. mikatae	0.12	0.67
sce00450	Selenocompound metabolism	S. mikatae	S. kudriavzevii	0.08	0.68
sce00480	Glutathione metabolism	S. cerevisiae	S. kudriavzevii	0.08	0.56
sce00062	Fatty acid elongation in mitochondria	S. cerevisiae	S. kudriavzevii	0.03	0.57
sce00565	Ether lipid metabolism	S. cerevisiae	S. kudriavzevii	-0.03	0.62
sce00970	Aminoacyl-tRNA biosynthesis	S. mikatae	S. kudriavzevii	-0.02	0.57
sce00750	Vitamin B6 metabolism	S. mikatae	S. kudriavzevii	-0.02	0.53
sce00730	Thiamine metabolism	S. paradoxus	S. kudriavzevii	-0.07	0.63
sce00450	Selenocompound metabolism	S. cerevisiae	S. kudriavzevii	-0.05	0.66
sce00500	Starch and sucrose metabolism	S. cerevisiae	S. paradoxus	-0.04	0.67
sce04070	Phosphatidylinositol signaling system	S. paradoxus	S. kudriavzevii	-0.06	0.50
sce03015	mRNA surveillance pathway	S. cerevisiae	S. kudriavzevii	-0.06	0.78
sce00860	Porphyrin and chlorophyll metabolism	S. cerevisiae	S. kudriavzevii	-0.10	0.65
sce00062	Fatty acid elongation in mitochondria	S. cerevisiae	S. mikatae	-0.11	0.70
sce00071	Fatty acid metabolism	S. mikatae	S. kudriavzevii	-0.15	0.63
sce00300	Lysine biosynthesis	S. cerevisiae	S. kudriavzevii	-0.16	0.68
sce00380	Tryptophan metabolism	S. mikatae	S. kudriavzevii	-0.15	0.67
sce00860	Porphyrin and chlorophyll metabolism	S. cerevisiae	S. paradoxus	-0.16	0.57
sce00630	Glyoxylate and dicarboxylate metabolism	S. cerevisiae	S. paradoxus	-0.16	0.65
sce00040	Pentose and glucuronate interconversions	S. paradoxus	S. kudriavzevii	-0.22	0.62
sce00510	N-Glycan biosynthesis	S. mikatae	S. kudriavzevii	-0.27	0.57
sce00510	N-Glycan biosynthesis	S. cerevisiae	S. kudriavzevii	-0.28	0.74
sce00410	beta-Alanine metabolism	S. cerevisiae	S. paradoxus	-0.29	0.67
sce00670	One carbon pool by folate	S. paradoxus	S. mikatae	-0.29	0.63
sce00340	Histidine metabolism	S. cerevisiae	S. mikatae	-0.31	0.61
sce00100	Steroid biosynthesis	S. cerevisiae	S. mikatae	-0.34	0.59
sce00680	Methane metabolism	S. paradoxus	S. kudriavzevii	-0.32	0.59
sce03015	mRNA surveillance pathway	S. mikatae	S. kudriavzevii	-0.35	0.54
sce00300	Lysine biosynthesis	S. paradoxus	S. kudriavzevii	-0.37	1.00
sce00520	Amino sugar and nucleotide sugar metabolism	S. paradoxus	S. kudriavzevii	-0.36	0.63
sce00062	Fatty acid elongation in mitochondria	S. mikatae	S. kudriavzevii	-0.39	0.59
sce04141	Protein processing in endoplasmic reticulum	S. paradoxus	S. mikatae	-0.40	0.67
sce00510	N-Glycan biosynthesis	S. cerevisiae	S. mikatae	-0.42	0.60
sce00780	Biotin metabolism	S. cerevisiae	S. kudriavzevii	-0.46	0.67

sce00500	Starch and sucrose metabolism	S. paradoxus	S. mikatae	-0.46	1.00
sce00250	Alanine, aspartate and glutamate metabolism	S. paradoxus	S. kudriavzevii	-0.51	0.33
sce00380	Tryptophan metabolism	S. cerevisiae	S. kudriavzevii	-0.52	0.67
sce00630	Glyoxylate and dicarboxylate metabolism	S. paradoxus	S. mikatae	-0.55	1.00
sce00562	Inositol phosphate metabolism	S. cerevisiae	S. paradoxus	-0.56	0.80
sce00970	Aminoacyl-tRNA biosynthesis	S. cerevisiae	S. kudriavzevii	-0.57	0.61
sce00900	Terpenoid backbone biosynthesis	S. paradoxus	S. mikatae	-0.57	0.75
sce00730	Thiamine metabolism	S. mikatae	S. kudriavzevii	-0.59	1.00
sce00910	Nitrogen metabolism	S. cerevisiae	S. paradoxus	-0.60	0.40
sce00790	Folate biosynthesis	S. paradoxus	S. kudriavzevii	-0.64	0.53
sce04130	SNARE interactions in vesicular transport	S. cerevisiae	S. kudriavzevii	-0.64	1.00
sce00480	Glutathione metabolism	S. cerevisiae	S. mikatae	-0.66	0.63
sce00071	Fatty acid metabolism	S. cerevisiae	S. mikatae	-0.68	0.83
sce00565	Ether lipid metabolism	S. mikatae	S. kudriavzevii	-0.56	0.69
sce00900	Terpenoid backbone biosynthesis	S. mikatae	S. kudriavzevii	-0.72	0.80
sce00680	Methane metabolism	S. mikatae	S. kudriavzevii	-0.78	1.00
sce00100	Steroid biosynthesis	S. cerevisiae	S. paradoxus	-0.79	0.57
sce00400	Phenylalanine, tyrosine and tryptophan biosynthesis	S. cerevisiae	S. mikatae	-0.80	1.00
sce00300	Lysine biosynthesis	S. mikatae	S. kudriavzevii	-0.75	0.50
sce03018	RNA degradation	S. mikatae	S. kudriavzevii	-0.81	0.62
sce00300	Lysine biosynthesis	S. cerevisiae	S. mikatae	-0.81	0.71
sce03018	RNA degradation	S. cerevisiae	S. kudriavzevii	-0.85	1.00
sce00920	Sulfur metabolism	S. paradoxus	S. kudriavzevii	-0.88	0.67
sce03013	RNA transport	S. paradoxus	S. kudriavzevii	-0.89	0.87
sce00730	Thiamine metabolism	S. cerevisiae	S. kudriavzevii	-0.90	0.50
sce00410	beta-Alanine metabolism	S. paradoxus	S. kudriavzevii	-0.93	0.78
sce00740	Riboflavin metabolism	S. paradoxus	S. kudriavzevii	-0.94	0.67
sce00900	Terpenoid backbone biosynthesis	S. cerevisiae	S. mikatae	-0.95	0.60
sce00100	Steroid biosynthesis	S. paradoxus	S. kudriavzevii	-0.93	0.67
sce00620	Pyruvate metabolism	S. cerevisiae	S. kudriavzevii	-0.97	0.56
sce00380	Tryptophan metabolism	S. cerevisiae	S. mikatae	-0.97	0.78
sce00562	Inositol phosphate metabolism	S. cerevisiae	S. mikatae	-0.97	0.69
sce04070	Phosphatidylinositol signaling system	S. mikatae	S. kudriavzevii	-1.01	0.74
sce04122	Sulfur relay system	S. mikatae	S. kudriavzevii	-0.95	0.50
sce00520	Amino sugar and nucleotide sugar metabolism	S. cerevisiae	S. kudriavzevii	-1.07	1.00
sce00600	Sphingolipid metabolism	S. paradoxus	S. kudriavzevii	-1.07	0.64
sce00900	Terpenoid backbone biosynthesis	S. cerevisiae	S. paradoxus	-1.08	0.77
sce00563	Glycosylphosphatidylinositol(GPI)-anchor biosynthesis	S. cerevisiae	S. kudriavzevii	-1.13	0.56
sce00380	Tryptophan metabolism	S. cerevisiae	S. paradoxus	-1.14	0.64
sce00650	Butanoate metabolism	S. paradoxus	S. mikatae	-1.11	0.63
sce00340	Histidine metabolism	S. paradoxus	S. mikatae	-1.16	0.92
sce00670	One carbon pool by folate	S. mikatae	S. kudriavzevii	-1.21	0.83
sce00770	Pantothenate and CoA biosynthesis	S. mikatae	S. kudriavzevii	-1.23	1.00
sce00600	Sphingolipid metabolism	S. cerevisiae	S. mikatae	-1.23	0.67
sce00410	beta-Alanine metabolism	S. mikatae	S. kudriavzevii	-1.28	0.44
sce00900	Terpenoid backbone biosynthesis	S. cerevisiae	S. kudriavzevii	-1.34	0.61
sce00970	Aminoacyl-tRNA biosynthesis	S. cerevisiae	S. mikatae	-1.31	0.74
sce00260	Glycine, serine and threonine metabolism	S. paradoxus	S. mikatae	-1.35	0.61
sce03013	RNA transport	S. cerevisiae	S. paradoxus	-1.37	0.50
sce00510	N-Glycan biosynthesis	S. paradoxus	S. mikatae	-1.34	0.79
sce03015	mRNA surveillance pathway	S. paradoxus	S. mikatae	-1.36	0.68
sce00600	Sphingolipid metabolism	S. mikatae	S. kudriavzevii	-1.37	0.69
sce00400	Phenylalanine, tyrosine and tryptophan biosynthesis	S. paradoxus	S. kudriavzevii	-1.46	0.77
sce00040	Pentose and glucuronate interconversions	S. cerevisiae	S. kudriavzevii	-1.33	0.65

sce00100	Steroid biosynthesis	S. cerevisiae	S. kudriavzevii	-1.43	0.76
sce00740	Riboflavin metabolism	S. paradoxus	S. mikatae	-1.53	0.70
sce00040	Pentose and glucuronate interconversions	S. mikatae	S. kudriavzevii	-1.37	0.67
sce00600	Sphingolipid metabolism	S. cerevisiae	S. kudriavzevii	-1.54	0.71
sce00563	Glycosylphosphatidylinositol(GPI)-anchor biosynthesis	S. cerevisiae	S. paradoxus	-1.55	0.52
sce00250	Alanine, aspartate and glutamate metabolism	S. mikatae	S. kudriavzevii	-1.64	0.33
sce04141	Protein processing in endoplasmic reticulum	S. mikatae	S. kudriavzevii	-1.58	0.64
sce00330	Arginine and proline metabolism	S. cerevisiae	S. mikatae	-1.65	0.55
sce04011	MAPK signaling pathway - yeast	S. paradoxus	S. mikatae	-1.66	0.61
sce04070	Phosphatidylinositol signaling system	S. cerevisiae	S. kudriavzevii	-1.66	0.67
sce00564	Glycerophospholipid metabolism	S. mikatae	S. kudriavzevii	-1.66	0.61
sce00250	Alanine, aspartate and glutamate metabolism	S. cerevisiae	S. kudriavzevii	-1.75	0.60
sce00730	Thiamine metabolism	S. paradoxus	S. mikatae	-1.68	0.90
sce00052	Galactose metabolism	S. cerevisiae	S. paradoxus	-1.76	0.50
sce00410	beta-Alanine metabolism	S. cerevisiae	S. kudriavzevii	-1.83	0.61
sce04011	MAPK signaling pathway - yeast	S. cerevisiae	S. paradoxus	-1.88	0.56
sce03015	mRNA surveillance pathway	S. cerevisiae	S. mikatae	-1.87	0.70
sce00562	Inositol phosphate metabolism	S. paradoxus	S. mikatae	-1.88	0.75
sce00030	Pentose phosphate pathway	S. paradoxus	S. kudriavzevii	-2.08	0.58
sce00760	Nicotinate and nicotinamide metabolism	S. paradoxus	S. mikatae	-2.07	0.60
sce00563	Glycosylphosphatidylinositol(GPI)-anchor biosynthesis	S. cerevisiae	S. mikatae	-2.03	0.55
sce00051	Fructose and mannose metabolism	S. cerevisiae	S. kudriavzevii	-2.24	0.57
sce04141	Protein processing in endoplasmic reticulum	S. cerevisiae	S. kudriavzevii	-2.17	0.71
sce00563	Glycosylphosphatidylinositol(GPI)-anchor biosynthesis	S. paradoxus	S. mikatae	-2.22	0.57
sce00380	Tryptophan metabolism	S. paradoxus	S. mikatae	-2.21	0.57
sce00330	Arginine and proline metabolism	S. paradoxus	S. kudriavzevii	-2.35	0.60
sce04011	MAPK signaling pathway - yeast	S. cerevisiae	S. mikatae	-2.34	0.70
sce00790	Folate biosynthesis	S. mikatae	S. kudriavzevii	-2.26	0.57
sce00770	Pantothenate and CoA biosynthesis	S. cerevisiae	S. mikatae	-2.39	0.63
sce00480	Glutathione metabolism	S. cerevisiae	S. paradoxus	-2.34	0.63
sce00330	Arginine and proline metabolism	S. paradoxus	S. mikatae	-2.42	0.60
sce00400	Phenylalanine, tyrosine and tryptophan biosynthesis	S. cerevisiae	S. kudriavzevii	-2.41	0.74
sce00600	Sphingolipid metabolism	S. paradoxus	S. mikatae	-2.33	0.58
sce00030	Pentose phosphate pathway	S. cerevisiae	S. paradoxus	-2.55	0.60
sce00564	Glycerophospholipid metabolism	S. paradoxus	S. mikatae	-2.44	0.64
sce00052	Galactose metabolism	S. paradoxus	S. mikatae	-2.52	0.50
sce00520	Amino sugar and nucleotide sugar metabolism	S. cerevisiae	S. mikatae	-2.53	0.63
sce00330	Arginine and proline metabolism	S. mikatae	S. kudriavzevii	-2.58	0.63
sce00051	Fructose and mannose metabolism	S. cerevisiae	S. mikatae	-2.62	0.63
sce00564	Glycerophospholipid metabolism	S. cerevisiae	S. kudriavzevii	-2.56	0.75
sce00740	Riboflavin metabolism	S. cerevisiae	S. kudriavzevii	-2.69	0.67
sce00480	Glutathione metabolism	S. paradoxus	S. kudriavzevii	-2.68	0.60
sce00520	Amino sugar and nucleotide sugar metabolism	S. mikatae	S. kudriavzevii	-2.77	1.00
sce00270	Cysteine and methionine metabolism	S. paradoxus	S. kudriavzevii	-2.93	0.63
sce00860	Porphyrin and chlorophyll metabolism	S. paradoxus	S. mikatae	-2.69	0.50
sce00770	Pantothenate and CoA biosynthesis	S. cerevisiae	S. paradoxus	-2.81	0.64
sce00400	Phenylalanine, tyrosine and tryptophan biosynthesis	S. mikatae	S. kudriavzevii	-2.84	1.00
sce00564	Glycerophospholipid metabolism	S. cerevisiae	S. paradoxus	-2.80	0.63

sce00740	Riboflavin metabolism	S. mikatae	S. kudriavzevii	-2.79	0.53
sce00480	Glutathione metabolism	S. paradoxus	S. mikatae	-2.82	0.67
sce00500	Starch and sucrose metabolism	S. cerevisiae	S. mikatae	-3.16	0.58
sce00770	Pantothenate and CoA biosynthesis	S. paradoxus	S. kudriavzevii	-2.98	0.58
sce00760	Nicotinate and nicotinamide metabolism	S. cerevisiae	S. mikatae	-3.19	0.67
sce00400	Phenylalanine, tyrosine and tryptophan biosynthesis	S. paradoxus	S. mikatae	-3.07	0.50
sce00010	Glycolysis / Gluconeogenesis	S. paradoxus	S. kudriavzevii	-3.37	0.57
sce00740	Riboflavin metabolism	S. cerevisiae	S. paradoxus	-3.22	0.80
sce04011	MAPK signaling pathway - yeast	S. paradoxus	S. kudriavzevii	-3.31	0.63
sce00670	One carbon pool by folate	S. cerevisiae	S. mikatae	-3.29	0.54
sce00250	Alanine, aspartate and glutamate metabolism	S. paradoxus	S. mikatae	-3.47	0.67
sce00520	Amino sugar and nucleotide sugar metabolism	S. paradoxus	S. mikatae	-3.44	0.52
sce00480	Glutathione metabolism	S. mikatae	S. kudriavzevii	-3.34	0.67
sce00860	Porphyrin and chlorophyll metabolism	S. mikatae	S. kudriavzevii	-3.25	0.58
sce03013	RNA transport	S. cerevisiae	S. kudriavzevii	-3.63	0.67
sce00250	Alanine, aspartate and glutamate metabolism	S. cerevisiae	S. paradoxus	-3.72	0.70
sce00030	Pentose phosphate pathway	S. cerevisiae	S. mikatae	-3.93	0.58
sce00330	Arginine and proline metabolism	S. cerevisiae	S. kudriavzevii	-3.73	0.73
sce00670	One carbon pool by folate	S. cerevisiae	S. kudriavzevii	-3.55	0.60
sce00670	One carbon pool by folate	S. cerevisiae	S. paradoxus	-3.67	0.68
sce00250	Alanine, aspartate and glutamate metabolism	S. cerevisiae	S. mikatae	-3.86	0.59
sce00051	Fructose and mannose metabolism	S. paradoxus	S. mikatae	-3.80	0.67
sce00860	Porphyrin and chlorophyll metabolism	S. cerevisiae	S. mikatae	-3.49	0.66
sce00620	Pyruvate metabolism	S. cerevisiae	S. paradoxus	-3.93	0.62
sce00920	Sulfur metabolism	S. paradoxus	S. mikatae	-3.40	0.65
sce00760	Nicotinate and nicotinamide metabolism	S. mikatae	S. kudriavzevii	-3.97	0.50
sce00680	Methane metabolism	S. cerevisiae	S. paradoxus	-3.97	0.57
sce04011	MAPK signaling pathway - yeast	S. mikatae	S. kudriavzevii	-3.96	0.67
sce00760	Nicotinate and nicotinamide metabolism	S. cerevisiae	S. kudriavzevii	-4.22	0.60
sce00052	Galactose metabolism	S. paradoxus	S. kudriavzevii	-4.09	0.63
sce00030	Pentose phosphate pathway	S. mikatae	S. kudriavzevii	-4.52	0.55
sce00030	Pentose phosphate pathway	S. paradoxus	S. mikatae	-4.46	0.58
sce00260	Glycine, serine and threonine metabolism	S. paradoxus	S. kudriavzevii	-4.13	0.55
sce00030	Pentose phosphate pathway	S. cerevisiae	S. kudriavzevii	-4.65	0.55
sce03008	Ribosome biogenesis in eukaryotes	S. cerevisiae	S. paradoxus	-4.34	0.75
sce03008	Ribosome biogenesis in eukaryotes	S. paradoxus	S. mikatae	-4.31	0.54
sce03008	Ribosome biogenesis in eukaryotes	S. cerevisiae	S. kudriavzevii	-4.43	0.50
sce00740	Riboflavin metabolism	S. cerevisiae	S. mikatae	-4.33	0.61
sce04011	MAPK signaling pathway - yeast	S. cerevisiae	S. kudriavzevii	-4.40	0.62
sce00020	Citrate cycle (TCA cycle)	S. cerevisiae	S. paradoxus	-4.87	0.54
sce03008	Ribosome biogenesis in eukaryotes	S. paradoxus	S. kudriavzevii	-4.62	0.63
sce00680	Methane metabolism	S. cerevisiae	S. kudriavzevii	-4.50	0.57
sce00052	Galactose metabolism	S. cerevisiae	S. mikatae	-4.56	0.60
sce00770	Pantothenate and CoA biosynthesis	S. cerevisiae	S. kudriavzevii	-4.67	0.56
sce00010	Glycolysis / Gluconeogenesis	S. paradoxus	S. mikatae	-5.30	0.75
sce00052	Galactose metabolism	S. mikatae	S. kudriavzevii	-4.87	0.67
sce00564	Glycerophospholipid metabolism	S. cerevisiae	S. mikatae	-4.34	0.67
sce00010	Glycolysis / Gluconeogenesis	S. cerevisiae	S. paradoxus	-5.42	0.60
sce00052	Galactose metabolism	S. cerevisiae	S. kudriavzevii	-4.95	0.58
sce00680	Methane metabolism	S. cerevisiae	S. mikatae	-5.00	0.56
sce00260	Glycine, serine and threonine metabolism	S. mikatae	S. kudriavzevii	-4.94	0.53
sce03008	Ribosome biogenesis in eukaryotes	S. cerevisiae	S. mikatae	-5.38	0.75
sce00260	Glycine, serine and threonine metabolism	S. cerevisiae	S. kudriavzevii	-5.30	0.61
sce03008	Ribosome biogenesis in eukaryotes	S. mikatae	S. kudriavzevii	-5.47	0.57

sce00270	Cysteine and methionine metabolism	S. cerevisiae	S. paradoxus	-6.21	0.72
sce00020	Citrate cycle (TCA cycle)	S. cerevisiae	S. mikatae	-6.28	0.56
sce00020	Citrate cycle (TCA cycle)	S. paradoxus	S. mikatae	-6.22	0.50
sce00620	Pyruvate metabolism	S. cerevisiae	S. mikatae	-5.87	0.80
sce00020	Citrate cycle (TCA cycle)	S. cerevisiae	S. kudriavzevii	-6.78	0.54
sce00270	Cysteine and methionine metabolism	S. cerevisiae	S. kudriavzevii	-6.95	0.65
sce00240	Pyrimidine metabolism	S. cerevisiae	S. paradoxus	-7.30	1.00
sce00010	Glycolysis / Gluconeogenesis	S. cerevisiae	S. mikatae	-9.48	0.59
sce00010	Glycolysis / Gluconeogenesis	S. cerevisiae	S. kudriavzevii	-7.45	0.63
sce00010	Glycolysis / Gluconeogenesis	S. mikatae	S. kudriavzevii	-8.45	0.60
sce00020	Citrate cycle (TCA cycle)	S. paradoxus	S. kudriavzevii	-7.74	0.67
sce00020	Citrate cycle (TCA cycle)	S. mikatae	S. kudriavzevii	-8.82	0.57
sce00240	Pyrimidine metabolism	S. paradoxus	S. mikatae	-7.98	0.55
sce00240	Pyrimidine metabolism	S. paradoxus	S. kudriavzevii	-9.81	0.56
sce00270	Cysteine and methionine metabolism	S. cerevisiae	S. mikatae	-7.43	0.56
sce00270	Cysteine and methionine metabolism	S. mikatae	S. kudriavzevii	-8.21	0.55
sce04111	Cell cycle - yeast	S. cerevisiae	S. paradoxus	-10.63	0.55
sce04111	Cell cycle - yeast	S. paradoxus	S. mikatae	-9.04	0.60
sce04111	Cell cycle - yeast	S. paradoxus	S. kudriavzevii	-11.59	0.62
sce04113	Meiosis - yeast	S. cerevisiae	S. paradoxus	-10.30	0.62
sce04113	Meiosis - yeast	S. paradoxus	S. mikatae	-8.26	0.60
sce04113	Meiosis - yeast	S. paradoxus	S. kudriavzevii	-10.65	0.58
sce04144	Endocytosis	S. paradoxus	S. mikatae	-1.00	0.64

3 Supplementary Text

3.1 Dynamical system model reconstruction

We present a general method to reconstruct dynamical system models (DSMs) from observed data. The method will obtain $\hat{f}_i$ with estimated parameters in the deterministic part f_i in the nonlinear additive ordinary differential equation (ODE) given in the main text. Let a sample of size n be taken at time $t_1, \ldots, t_n$.

Given a mathematical form of f_i with unknown parameters, we obtain an estimate $\hat{f}_i$ for variable i to minimize residuals by least squares. Then we measure its goodness-of-fit using the residual sum of squares (RSS), by

$$RSS(i) = \sum_{k=1}^{n} \left[y_i(t_k) - \hat{f}_i(\mathbf{x}(t_k)) \right]^2 \tag{S1}$$

We also define a null model $\hat{f}_i^0$, embedded in and simpler than f_i, by time average of $y_i(t_k)$ as

$$\hat{f}_i^0 = \frac{1}{n} \sum_{k=1}^{n} y_i(t_k) \tag{S2}$$

Similarly, we compute the residual sum of squares $RSS_0(i)$ for $\hat{f}_i^0$. $RSS(i)$ is compared with $RSS_0(i)$ to quantify the improvement by $\hat{f}_i$ over $\hat{f}_i^0$ using Fisher's F-test

$$F(i) = \frac{[RSS_0(i) - RSS(i)]/u}{RSS(i)/v} \tag{S3}$$

where $u = m(i) - m_0(i)$ and $v = n - m(i)$ are the degrees of freedom with $m_0(i) = 1$ being the model complexity (number of parameters) of $\hat{f}_i^0$ and $m(i)$ the model complexity of $\hat{f}_i$. Under standard normality assumptions, $F(i)$ follows an F-distribution with u-numerator and v-denominator degrees of freedom under the null hypothesis of $\hat{f}_i^0$ being the true model.

To choose the mathematical form and optimize parameters of model $\hat{f}_i$ for variable i, we enumerate the linear combination of the nonlinear terms and select one with the most significant improvement by minimizing the p-value computed for $F(i)$

$$\hat{f}_i = \operatorname*{argmin}_{f_i} \{p\text{-value of } F(i) \text{ with } f_i\} \tag{S4}$$

The above outlines a basic regression method to estimate f_i, and it is possible to use other strategies more pertinent to a data set. For example, in comparative dynamical system modeling, the F-statistic used is computed on multiple data sets under different conditions as described in the main text.

Our DSM, employing additive linear, quadratic, and sigmoidal ODEs, has biases towards such parametric forms. It may be, however, less critical to model comparison than model reconstruction. Despite using inexact ODE forms from most BioModels (Chelliah et al., 2013), DSM approximation demonstrated a considerable improvement over linear approximation used in GSCA in pathway comparison. Additionally, we assume availability of sufficiently sampled time course data to estimate derivatives accurately. Also, as we assume a same topology for both conditions, pathway rewiring is implicitly achieved by close-to-zero coefficients for some interactions in one condition but not the other. Finally, finding a sub-graph from a given pathway topology, adaptation of known interactions is facilitated, but not for novel interactions, which can be complemented by interaction-driven comparative dynamical system modeling (Ouyang et al., 2011).

3.2 Comparable performance of GSCA and the Q-method at various noise levels

We generated ground truth of conserved and differential models from original models in BioModels database (Chelliah et al., 2013). A conserved model is the same with an original model but with a changed initial state. This can have a dramatic effect on system dynamics. The initial value of a variable was changed by adding a random number uniformly distributed in $[L, U]$ to the original initial state. L and U are determined by model sensitivity to the initial state. Too much change can break a relationship between variables due to numerical instability.

To create a differential model, parameter values in the original model are changed by a random number normally distributed with mean μ and standard deviation σ. To avoid numerical instability, μ and σ vary depending on model sensitivity to parameters.

To cover diverse initial states and parameters, we generated 100 conserved and 100 differential models for each original model. Given a model and its initial state, we simulated time courses and added noises at signal-to-noise ratios (SNRs) of 100, 20, 10, or 0 dB. The time courses are generated by solving ODEs in each model using R package `deSolve` Soetaert et al. (2010), and sampled at 100 time points, uniformly spaced with a model-dependent time increment between each pair of time points. The perturbation values for each model are described in main text Table 1, and the runtime of the simulation study on each model is given in main text Table 2.

Outstanding from most BioModels (Chelliah et al., 2013) on which we tested, the Q-method and GSCA performed comparably on a circadian oscillation model (BIOM0000000021) at a 100 dB signal-to-noise ratio (SNR) in Figure S5. The similar performance of the two methods can be explained by Figure S6, showing the phase planes of each pair

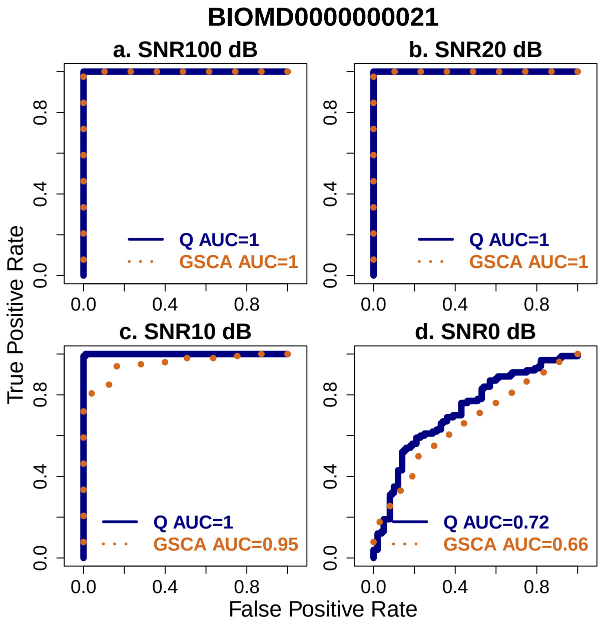

Figure S5. The ROC curves by the Q-method (blue lines) and GSCA (orange dots) for a circadian oscillation model (BioModels BIOM0000000021) at SNRs of 100, 20, 10, and 0 dB. GSCA performed worse than but comparably to the Q-method.

of nodes on the BIOM0000000021 pathway. In the figure, pairwise linear correlation in GSCA is consistently high between the apparently similar trajectories generated by the original and the conserved models, but very weak due to distinct differences between the trajectories of the original and the differential models. This situation is likely to arise when a conserved model starts at an initial state very similar to the original model – critical for GSCA to work effectively on non-chaotic nonlinear systems.

3.3 Predicting rewired pathways among four yeast species

To determine whether inherent genetic variations can be predicted by detected pathway heterogeneity using the Q-method, we applied the method to compare transcriptome time courses from four different yeast species: *S. cerevisiae*, *S. paradoxus*, *S. mikatae*, and *S. kudriavzevii*. We first obtained pathway heterogeneity for each of the 68 yeast pathways in KEGG between each pair of the four yeast species using their transcriptomes under various stress conditions (Tirosh et al., 2006). Using their respective reference genome sequences, we further calculated TATA box disparity between pathway pairs among the four yeast species. We finally report a strong association between pathway interaction heterogeneity and pathway TATA box disparity among the four species.

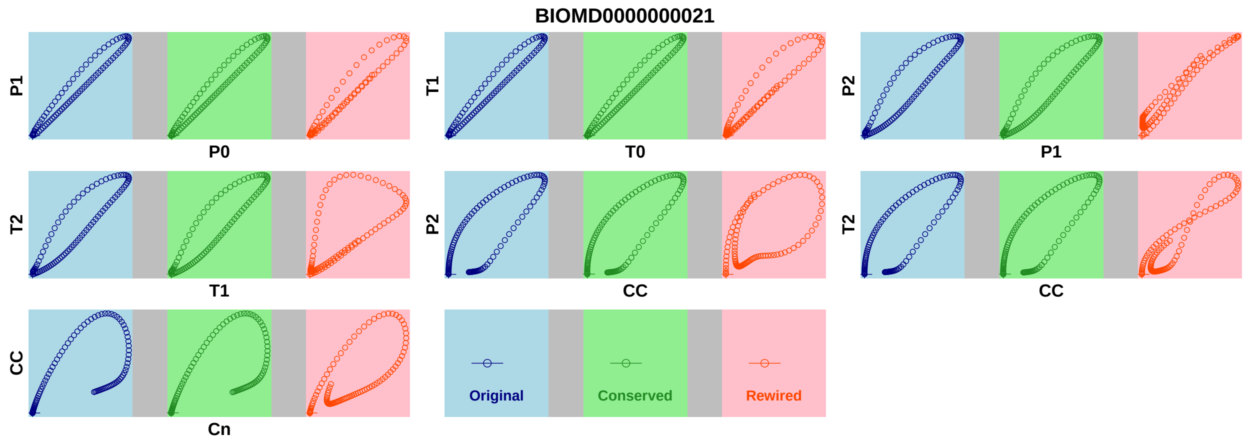

Figure S6. Dynamic behavior conducive to both Q-method and GSCA on comparing a circadian oscillation model (BIOM0000000021). Phase planes in each grey box are the interactions between nodes on the pathway in the original (blue), conserved (green), and rewired/differential (red) models. The original and conserved phase planes show very little change, but the original and differential differ substantially among the pairs of P1-P2, T1-T2, P2-CC, and T2-CC.

3.3.1 TATA box detection

The reference genomes of the four yeast species were published by Engel et al. (2014) for *S. cerevisiae*, by Kellis et al. (2003) for *S. paradoxus* and *S. mikatae*, and by Cliften et al. (2003) for *S. kudriavzevii*. The open reading frames (ORFs) and all reference yeast genomes were downloaded from *Saccharomyces* Genome Database (`www.yeastgenome.org`). We first annotated each genome other than the already annotated *S. cerevisiae*. For each ORF in *S. cerevisiae*, we find its match within each of the three other genomes using BLAST (Altschul et al., 1990). For each identified occurrence of an ORF, the 300bp sequence region upstream of the ORF's start codon was extracted. Then we searched for TATA boxes in these upstream regions using the consensus sequence motif TATA(A/T)A(A/T)(A/G) reported for yeast by (Basehoar et al., 2004). The number and percentage of TATA-containing genes are summarized in Table S5. Our result shows that TATA box presence in a genome is weakly conserved among the yeast species.

Table S3. Statistics on TATA-containing genes in four yeast species. For each species, the total number of gene in its genome, and the number and percentage of TATA box-containing genes are given.

Species	Number of ORFs	TATA-containing	Percentage
Saccharomyces cerevisiae	5044	860	17.05%
Saccharomyces paradoxus	4942	909	18.39%
Saccharomyces mikatae	3954	650	16.44%
Saccharomyces kudriavzevii	3400	399	11.74%

The number of genes containing a TATA box in their promoters is found to be a little less than 20% in three of the four studied yeast species. *S. paradoxus* has the most abundance of TATA box-containing genes. However, *S. kudriavzevii* has the least percentage of TATA-containing genes.

3.3.2 Pathway TATA box disparity

We define pathway TATA box disparity between two species as the ratio of the number of genes in that pathway with TATA box present in only one species to the number of genes with a TATA box in either one of the two species. That

is

$$\text{Pathway TATA box disparity} = \frac{\text{Number of genes TATA-containing in one species but TATA-less in the other within the pathway}}{\text{Number of TATA-containing genes in either species within the pathway}} \quad \text{(S5)}$$

Table S6 illustrates how to compute pathway TATA box disparity. The pathway TATA box disparity column in Table S4

Table S4. An example to compute pathway TATA box disparity. We assume a pathway contains five genes. For the two species under comparison, the absence/presence of a TATA box on each gene is given in the table.

Pathway genes	TATA box in species 1	TATA box in species 2
Gene 1	absent	absent
Gene 2	present	present
Gene 3	absent	present
Gene 4	present	absent
Gene 5	absent	absent

The number of genes containing a TATA box in only one species is 2, and the number of genes containing a TATA box in either one of the two species is 3. We define the pathway TATA box disparity to be $2/3 = 0.67$.

reports the statistic on all 68 pathways compared across the four yeast species, ranging from 0.33 to 1.

3.3.3 Normalized pathway interaction heterogeneity

To satisfy the conditions for linear regression of pathway heterogeneity on pathway TATA box disparity shown in the main text, we normalized the Q_d to Q_d^*-statistic that is comparable across pathways with different degrees of freedom. Additionally, the linear regression residual qualitatively follows a normal distribution for the model significance to be evaluated using an F-distribution.

After computing the pathway heterogeneity Q-statistic using the expression data of four yeast species under various stresses (Tirosh et al., 2006), we normalized the Q_d-statistics from Q-method to Q_d^* for each pathway between two species using the following transformation (Laubscher, 1960):

$$Q_d^* = \frac{\sqrt{2v_d - 1} \cdot \sqrt{u_d Q_d / v_d} - \sqrt{2u_d - 1}}{\sqrt{u_d Q_d / v_d + 1}} \quad \text{(S6)}$$

where Q_d is the original pathway F-statistic, and u_d and v_d are the numerator and denominator degrees of freedom defined in the main text, respectively.

The normalized pathway heterogeneity and TATA box disparity for all 68 pathways across all 6 pairs of species are shown in Table S4. The scatterplot of these two measures is shown in Figure 6 in the main text. A linear regression analysis shows that normalized pathway heterogeneity Q_d^* and pathway TATA box disparity have a strong association with Pearson correlation $r = 0.47$ at a statistical significance level of $P = 1.09 \times 10^{-23}$.

3.3.4 Pathway interaction heterogeneity is consistent with TATA box disparity among yeast species

It has been shown that RNA polymerase (RNAP) II and III both interact with the TATA box as a basal level promoter element (Wang et al., 1996; Moshonov et al., 2008; Rhee and Pugh, 2012; Savinkova et al., 2013). Wang et al. (1996) also showed that point mutations within TATA box sequences directly affected the affinity for RNAP II and III. Furthermore the pattern of nucleotides of the first five positions may be responsible for RNAP selection, as RNAPIII demonstrated higher affinity for tandem T residues and RNAPII showed a higher affinity for alternating T-A residues. TATA box-binding proteins (TBPs) which associate the TATA box with RNAP have been shown to affect transcription

levels (Shen et al., 1998). The TBP Brf in yeast promotes transcription of the U6 snRNA gene, and when a loss of function Brf mutant is introduced transcription by RNAPII drops to basal levels (Shen et al., 1998). While transcription is not inhibited by a loss of function TBP or absence of a TATA box it can be reduced to basal levels as stability of the RNAP promoter complex is reduced.

The association between normalized pathway heterogeneity computed from the transcriptomes and pathway TATA box disparity from the genomes between species differed among pathways, but showed a statistically significant positive correlation (Figure 6 in the main text). Some pathways such as alanine, aspartate, and glutamate metabolism have both low pathway heterogeneity and low pathway TATA box disparity. Nitrogen metabolism showed a remarkable positive correlation between normalized pathway heterogeneity and pathway TATA box disparity. Pathways with low heterogeneity may not be similar due to necessity but rather have not had sufficient time to diverge. Many pathways observed displayed a 100% TATA box disparity like various types of N-glycan biosynthesis and folate biosynthesis with heterogeneity values of 10.68 and 6.95, respectively. It is possible that some pathways are more plastic than others in regards to variations in promoter regions and gene expression, while others are more rigid and under strict purifying selection. While high pathway heterogeneity and high pathway TATA box disparity is a trend generally observed, it is interesting to note how pathways such as nitrogen metabolism, between two species can be highly conserved or divergent and the opposite is true between two other species. Therefore, similarities between the same pathway in two species may not be the result of purifying selection but rather a lack of divergence.

However, some highly conserved pathways such as glycolysis and gluconeogenesis with low heterogeneity (-9.48) showed a relatively high TATA box disparity (59%) among TATA-containing genes and were considered outliers. Glycolysis and gluconeogenesis are vital metabolic pathways necessary for the regulation of glucose metabolism and anabolism. It appears that TATA boxes in the genes in these pathways are not conserved among the species. This suggests that other expression regulatory elements, for example, evolution of DNA-binding domains of transcription factors (Babu et al., 2004), and evolution of regulatory DNA motifs (Gerland and Hwa, 2002), may be involved which maintain similar expression levels between species and may explain such outliers. The citric acid cycle (TCA), another vital metabolic pathway responsible for the generation of NADH and FADH2 for the electron transport chain, and generation of ATP, appeared to have relatively low heterogeneity (-4.43 to -8.82) and moderate TATA box disparity (50-67%). These discrepancies imply that pathway conservation does not always directly depend on TATA box conservation, and other regulatory elements may compensate for variations in TATA boxes to maintain expression integrity within a pathway.

A general trend of high heterogeneity of expression and high TATA box disparity can be seen when all pathways are compared. Outliers indicate that while strict purifying selection may exist for some genes, others have a higher degree of promoter region plasticity and the presence or absence of a TATA box may not accurately predict expression. While *in silico* expression prediction cannot be determined solely on the presence or absence of a TATA box, there is a correlation which lends confidence to such a prediction.

The TATA box disparity observed is consistent with the conclusion in (Tirosh et al., 2006) that as species diverge TATA box disparity increases. The expression levels of homologs between species varied as did the presence of TATA box regions. Tirosh et al. (2006) concluded that variability within promoter regions is the driving force of the evolution of gene expression variability. While the pathways themselves have remained relatively conserved, expression levels between species are variable as are their promoter regions. The correlation between low heterogeneity and low TATA box disparity while not always consistent implies that some genes may rely on a TATA box more than others for expression above basal levels. Differential gene expression between species may be partially explained by pathway rewiring through TATA box variations in presence, sequence, and position near the promoter and offer a possible mechanism for explaining phenotypic diversity among species.

References

Altschul, S. F., Gish, W., Miller, W., Myers, E. W., and Lipman, D. J. (1990). Basic local alignment search tool. *J. Mol. Biol.*, 215(3):403–410.

Babu, M. M., Luscombe, N. M., Aravind, L., Gerstein, M., and Teichmann, S. A. (2004). Structure and evolution of transcriptional regulatory networks. *Current Opinion in Structural Biology*, 14(3):283–291.

Basehoar, A. D., Zanton, S. J., and Pugh, B. (2004). Identification and distinct regulation of yeast TATA box-containing genes. *Cell*, 116(5):699 – 709.

Borghans, J. M., Dupont, G., and Goldbeter, A. (1997). Complex intracellular calcium oscillations. A theoretical exploration of possible mechanisms. *Biophys Chem*, 66:25–41.

Chelliah, V., Laibe, C., and Novère, N. L. (2013). BioModels database: a repository of mathematical models of biological processes. *Methods Mol. Biol.*, 1021:189–199.

Cliften, P., Sudarsanam, P., Desikan, A., Fulton, L., Fulton, B., Majors, J., Waterston, R., Cohen, B. A., and Johnston, M. (2003). Finding functional features in *Saccharomyces* genomes by phylogenetic footprinting. *Science*, 301(5629):71–76.

Engel, S. R., Dietrich, F. S., Fisk, D. G., Binkley, G., Balakrishnan, R., Costanzo, M. C., Dwight, S. S., Hitz, B. C., Karra, K., Nash, R. S., et al. (2014). The reference genome sequence of *Saccharomyces cerevisiae*: Then and now. *G3*, 4(3):389–398.

Fung, E., Wong, W. W., Suen, J. K., Bulter, T., Lee, S. G., and Liao, J. C. (2005). A synthetic gene-metabolic oscillator. *Nature*, 435:118–122.

Fuss, H., Dubitzky, W., Downes, S., and Kurth, M. J. (2006). Bistable switching and excitable behaviour in the activation of Src at mitosis. *Bioinformatics*, 22:158–165.

Gardner, T. S., Dolnik, M., and Collins, J. J. (1998). A theory for controlling cell cycle dynamics using a reversibly binding inhibitor. *Proc Natl Acad Sci U S A*, 95:14190–14195.

Gerland, U. and Hwa, T. (2002). On the selection and evolution of regulatory DNA motifs. *Journal of Molecular Evolution*, 55(4):386–400.

Hynne, F., Danø, S., and Sørensen, P. G. (2001). Full-scale model of glycolysis in *Saccharomyces cerevisiae*. *Biophys Chem*, 94:121–163.

Kellis, M., Patterson, N., Endrizzi, M., Birren, B., and Lander, E. S. (2003). Sequencing and comparison of yeast species to identify genes and regulatory elements. *Nature*, 423(6937):241–254.

Laubscher, N. F. (1960). Normalizing the noncentral t and F distributions. *Ann. Math. Statist.*, 31(4):835–1254.

Leloup, J. and Goldbeter, A. (1999). Chaos and birhythmicity in a model for circadian oscillations of the PER and TIM proteins in *Drosophila*. *Journal of Theoretical Biology*, 198:445–459.

Leloup, J. C. and Goldbeter, A. (2003). Toward a detailed computational model for the mammalian circadian clock. *Proc Natl Acad Sci U S A*, 100:7051–7056.

Moshonov, S., Elfakess, R., Golan-Mashiach, M., Sinvani, H., and Dikstein, R. (2008). Links between core promoter and basic gene features influence gene expression. *BMC Genomics*, 9(1):92.

Nielsen, K., Sørensen, P. G., Hynne, F., and Busse, H. G. (1998). Sustained oscillations in glycolysis: an experimental and theoretical study of chaotic and complex periodic behavior and of quenching of simple oscillations. *Biophys Chem*, 72:49–62.

Ouyang, Z., Song, M., Güth, R., Ha, T. J., Larouche, M., and Goldowitz, D. (2011). Conserved and differential gene interactions in dynamical biological systems. *Bioinformatics*, 27(20):2851–2858.

Rhee, H. S. and Pugh, B. F. (2012). Genome-wide structure and organization of eukaryotic pre-initiation complexes. *Nature*, 483(7389):295–301.

Savinkova, L., Drachkova, I., Arshinova, T., Ponomarenko, P., Ponomarenko, M., and Kolchanov, N. (2013). An experimental verification of the predicted effects of promoter TATA-box polymorphisms associated with human diseases on interactions between the TATA boxes and TATA-binding protein. *PLoS ONE*, 8(2):e54626.

Shen, Y., Kassavetis, G. A., Bryant, G. O., and Berk, A. J. (1998). Polymerase Pol III TATA box-binding protein (TBP)-associated factor Brf binds to a surface on TBP also required for activated Pol II transcription. *Mol. Cell. Biol.*, 18(3):1692–1700.

Soetaert, K., Petzoldt, T., and Setzer, R. W. (2010). Solving differential equations in R: package deSolve. *J. Stat. Softw.*, 33(9):1–25.

Tirosh, I., Weinberger, A., Carmi, M., and Barkai, N. (2006). A genetic signature of interspecies variations in gene expression. *Nat. Genet.*, 38(7):830–834.

Tyson, J. J. (1991). Modeling the cell division cycle: cdc2 and cyclin interactions. *P. Natl. Acad. Sci. USA*, 88:7328–7332.

Vilar, J. M., Kueh, H. Y., Barkai, N., and Leibler, S. (2002). Mechanisms of noise-resistance in genetic oscillators. *Proc Natl Acad Sci U S A*, 99:5988–5992.

Wang, Y., Jensen, R. C., and Stumph, W. E. (1996). Role of TATA box sequence and orientation in determining RNA polymerase II/III transcription specificity. *Nucleic Acids Res.*, 24(15):3100–3106.
